# Intranasal trivalent candidate vaccine elicits broad humoral and cellular immunity against pneumococcal pneumonia

**DOI:** 10.3389/fcimb.2025.1563661

**Published:** 2025-06-27

**Authors:** Fangyu Ren, Luyun Huang, Shilu Luo, Changjin Liu, Xianlian Chen, Xin Yao, Qiqi Linghu, Huaqin Hu, Xiaoyu Huang, Yuanqin Hu, Jian Huang, Xun Min

**Affiliations:** ^1^ Department of Laboratory Medicine, Affiliated Hospital of Zunyi Medical University, Zunyi, Guizhou, China; ^2^ School of Laboratory Medicine, Zunyi Medical University, Zunyi, Guizhou, China

**Keywords:** *Streptococcus pneumoniae*, trivalent vaccine, humoral immunity, cellular immunity, protective efficacy

## Abstract

*Streptococcus pneumoniae* is an important pathogen causing public health problems worldwide. Existing pneumococcal vaccines provide protection against only a few of the more than 100 pneumococcal serotypes, highlighting the urgent need for new preventive strategies. Pneumococcal protein vaccines have attracted considerable attention owing to their favorable immunogenicity and antigen conservation, and have demonstrated protective potential against non-serotype-dependent infections. Mice immunized with a trivalent vaccine targeting protein PepN, PepO, and SPD_1609 elicited a robust humoral immune response, as well as Th1, Th2, and Th17 cellular immune responses. The antiserum derived from the trivalent vaccine significantly inhibited *Streptococcus pneumoniae* adhesion to A549 cells, reduced pneumococcal colonization in the nasopharynx, and improved lung tissue damage and inflammatory responses compared to the monovalent or bivalent vaccine group. In terms of *in vivo* protection, the trivalent vaccine significantly increased the survival rate of infected mice. The findings suggest that the trivalent vaccine targeting PepN, PepO, and SPD_1609 is a promising multivalent vaccine candidate against *Streptococcus pneumoniae*.

## 1 Introduction

*Streptococcus pneumoniae* (*S. pneumoniae)* infections mainly cause diseases such as pneumonia, meningitis, and otitis media (Majumder et al., 2024) and are associated with a high mortality rate, particularly among children under 5 years old and adults 65 years and older. According to the World Health Organization, nearly one million children deaths are attributed to *S. pneumoniae* infections, with 50% of these fatalities occurring in the under-5 population annually, predominantly in developing countries (Wang et al., 2017). Antibiotics are the primary treatment option for *S. pneumoniae* infections. However, the prevalence of antimicrobial resistance is on the rise, with regional variations observed in this trend. Between 2012 and 2017, 81.2% of the drug-resistant strains of invasive pneumococcal disease in children were isolated in Beijing, China, predominantly exhibiting resistance to erythromycin, clindamycin, and tetracycline (Li et al., 2023a), The current antibiotic-based therapeutic strategies are facing severe challenges in combating infections caused by *S. pneumoniae*. Notably, the introduction of PCV7 and PCV13 vaccines resulted in decreased resistance to penicillin and erythromycin in vaccine-covered serotype strains, showing the effectiveness of the vaccine in controlling resistant strains. However, it was also found that penicillin-insensitivity of non-covered serotypes strains appeared rapidly. Therefore, the development of a more broad-spectrum vaccine may reduce the problem of new drug resistance caused by serotype conversion (Bajema et al., 2022; Sempere et al., 2022).

The current commercially available *S. pneumoniae* vaccines comprise polysaccharide and polysaccharide protein conjugate vaccines. 23-valent pneumococcal polysaccharide vaccines (PPV23) covers serotypes 1, 2, 3, 4, 5, 6B, 7F, 8, 9n, 9v, 10A, 11A, 12F, 14, 15B, 17F, 18C, 19A, 19F, 20, 22F, 23F, and 33F, which is poorly protective in young children under 2-years-of-age because of its weak antigenicity and non-T cell-dependent immunity. PPV23 is mainly appropriate for middle-aged and older people, and adults at high risk (Kraicer-Melamed et al., 2016). Polysaccharide protein conjugate vaccines, such as PCV7/10/13, are strongly antigenic and induce T cell-dependent immunity, providing better protection for the elderly and infants (Prato et al., 2018). Although PPV23 and PCV have been included in the *S. pneumoniae* vaccination program, frequent serotype substitutions, low serotype coverage, and an increase in non-encapsulated *S. pneumoniae* have contributed to the high mortality rates caused by *S. pneumoniae* infections (Masomian et al., 2020). Therefore, developing broader-spectrum pneumococcal vaccines—such as those targeting currently non-vaccine serotypes (e.g., 11A、22F、33F)—represents a critical priority for overcoming serotype replacement and antimicrobial resistance.

The *S. pneumoniae* protein vaccine has become a research hotspot for the development of vaccines against non-vaccine serotypes because of its good immunogenicity, conserved antigenicity, low cost, and ability to induce CD4^+^ T cell response (Lagousi et al., 2019). *S. pneumoniae* virulence factors and their surface-expressed proteins that effectively induce mucosal and serum antibody responses to block pathogenic infections are potential targets for vaccine candidates (Dagan, 2009). Pneumococcal lysine (PLY), pneumococcal surface protein A (PspA), and pneumococcal histidine triad proteins (Pht) have been implicated as vaccine candidates (Turner et al., 2017). A trivalent vaccine consisting of PspA, PLY derivative (dPly) and pneumococcal surface adhesion protein (PcpA) has demonstrated safety and tolerability in Phase I clinical trials (Scott et al., 2021; Musher et al., 2022; Wang et al., 2024). However, as there are more than 100 serotypes pneumococcal serotypes, it is challenging for a single protein antigen to induce a stable immune response and provide broad protection against multiple serotypes. To improve vaccine coverage, the use of multivalent vaccines consisting of more proteins capable of providing broad cross-protection independent of serotype has become an important strategy for the development of a new generation of vaccines against non-vaccine serotypes of *S. pneumoniae* (Scott et al., 2021). The Pn-MAPS24v vaccine, developed using the Multiple Antigen Presenting System (MAPS) platform, combines 24 *S. pneumoniae* serotype-specific polysaccharides (PS) with the conserved fusion protein CP1, forming macromolecular complexes. This design elicits both B-cell activation and T-cell-mediated immunity, potentially extending protection to non-vaccine serotypes. Currently, Pn-MAPS24v is undergoing Phase I clinical trials (Zhang et al., 2013; Borys et al., 2024).

SPD_1609 is a novel iron transporter protein that is highly conserved and immunogenic. This protein, which is mainly located in the cytoplasmic membrane of cells (Akhtar and Turner, 2022), is important for *S. pneumoniae* growth and iron uptake under iron-deficient conditions. SPD_1609 plays a key role in *S. pneumoniae* nasopharyngeal colonization, respiratory mucosal attachment, and host invasion (Yang et al., 2016, 2019). Aminopeptidase N (PepN) is a novel virulence factor of *S. pneumoniae* with strong immunogenicity. As a cell wall-localized protein, PepN can inhibit the effector function of cytotoxic T lymphocytes by modulating the intracellular T cell receptor signaling cascade, thereby reducing the production of the pro-inflammatory cytokine IFN-γ (Blevins et al., 2017). In addition, PepN enhances neutrophil elastase-mediated antimicrobial effects and is important in the adhesion and colonization of *S. pneumoniae* (Nganje et al., 2019). Endopeptidase O (PepO) is a metalloendopeptidase that is homologous to the M13 family of peptidases. PepO can induce a strong innate immune response through the Toll-like receptor 2 (TLR2)/TLR4 dual ligand-dependent pathway (Zhang et al., 2016), and PepO as a TLR2/TLR4 dual ligand could significantly enhance the non-specific phagocytic function and bactericidal activity of macrophages by inducing autophagy (Shu et al., 2020).

Based on the foregoing knowledge, we constructed a novel trivalent protein vaccine consisting of rPepN, rPepO, and rSPD_1609, and investigated whether the vaccine could stimulate effective mucosal and systemic immunity by intranasal immunization of mice to prevent lung inflammation caused by *S. pneumoniae.*

## 2 Materials and methods

### 2.1 Strains and culture conditions

*S. pneumoniae* D39 (serotype 2), CMCC(B)31693 (serotype 19F), CMCC31203 (serotype 3), and CMCC31207 (serotype 6B) were all derived in a previous study of our group (Wang et al., 2022), In addition, six *S. pneumoniae* strains obtained from clinical isolates at the Medical Laboratory Department of the Affiliated Hospital of Zunyi Medical University were included. These ten strains were inoculated on blood agar plates (BAP) and cultured overnight at 37°C in an atmosphere of 5% CO_2_. The next day, individual colonies were selected and transferred to Todd Hewitt Yeast (THY) broth medium to multiply bacteria, and when OD_600 nm_ reached 0.6, the bacterial pellet was harvested and the bacterial suspension was prepared with phosphate buffered saline (PBS).

### 2.2 Mice

Specific pathogen-free, BALB/c, female, six-eight-week-old mice purchased from the Experimental Animal Center of Zunyi Medical University (Zunyi, China) were maintained in the animal house of the Public Animal Laboratory Center, Zunyi Medical University. All animal experiments complied with the guidelines and codes of the Animal Experiment Ethics Committee of Zunyi Medical University (approval number: ZYFY-AN-2024-0668).

### 2.3 Immunization of mice

The *pepn*, *pepo* and *spd_1609* genes were cloned from the genomic DNA of *S. pneumoniae* strain D39. These recombinant proteins were expressed in *Escherichia coli* BL21 (DE3). Recombinant proteins were purified using a Ni^2+^-nitrilotriacetic acid column and the purity was ≥90%. Endotoxin was detected using the endotoxin detection kit provided by the same company. The endotoxin content of the final protein sample was maintained below 0.1 EU/mL. The mice were randomly divided into 8 groups: PepN, PepO, SPD_1609, PepN + PepO, PepN + SPD_1609, PepO + SPD_1609, PepN + PepO + SPD_1609 (Trivalent), and control (n = 8 per group). On days 0, 14, and 28, intranasal inoculations were performed with 10 µg of monovalent proteins (PepO, PepN, or SPD_1609), 20 µg of bivalent proteins (PepN + PepO, PepN + SPD_1609, or PepO + SPD_1609), or 30 µg of trivalent protein (PepN + PepO + SPD_1609). The proteins were dissolved in 20 µL of PBS and mixed with 5 µL of cholera toxin B subunit adjuvant (Absin, Shanghai, China), resulting in a final intranasal inoculation volume of 25 µL per mouse. Positive control mice received 100 µL of 23-valent pneumococcal polysaccharide vaccine (PPV23 vaccine, Changzheng Hospital, Honghuagang District, Zunyi, China) on the primary immunization. On the 14th day after the last immunization, the cardiac blood was collected for the subsequent passive protection test.

### 2.4 Antibody titer determination by enzyme-linked immunosorbent assay

Sera were collected from the immunized mice on days 7, 21 and 35 after the first immunization. Bronchoalveolar lavage fluid (BALF) was collected on day 42 after the first immunization. Antibody titers were determined by ELISA. Individual protein antigens were diluted to a final concentration of 10 µg/mL. Subsequently, 100 µL aliquot of the antigen solutions were dispensed into each wells and incubated overnight at 4°C. Wells of multiwell plates were incubated with PBS containing 2% bovine serum albumin for 2 h. After three washes with PBS containing 0.1% Tween 20 (PBST), the antisera of the immunized groups were diluted in a gradient manner, and incubated at 37°C for 1 h. After washing three times with PBST, horseradish peroxidase (HRP)-labeled goat anti-mouse IgG, IgG1, IgG2a and IgA diluted 1:5000 were added and incubated for 1 h at 37°C. The reaction was terminated by adding stop solution (Solarbio, Beijing, China) after 30 min of incubation with 3,3 ‘,5,5 ‘-tetramethylbenzidine (TMB) in the dark. Finally, absorbance was measured at 450 _nm_. The A_450 nm_ value of the sample was 2.1-fold higher than that of the negative control, which was defined as the highest antibody titers.

### 2.5 Western blot analysis of antiserum recognition of naturally expressed target proteins in *S. pneumoniae*

*S. pneumoniae* D39, 19F, CMCC31207 (serotype 6B), CMCC31203 (serotype 3) and four randomly collected clinical *S. pneumoniae* strains were inoculated on BAP and cultured overnight at 37°C in an atmosphere of 5% CO_2_. The next day, colonies was collected and washed with PBS. Loading buffer (1%) was added and boiled at 100°C for 15 min to denature the proteins. The bacterial lysates were subjected to separation using 10% sodium dodecyl sulfate-polyacrylamide gel electrophoresis (SDS-PAGE) and transferred to polyvinylidene fluoride membranes (Merck Millipore, Burlington, MA, USA). Mouse antiserum (each diluted 1:1000) against PepN, PepO, SPD_1609 and trivalent vaccine were used as primary antibodies, and the secondary antibody used was horseradish peroxidase (HRP)-conjugated goat anti-mouse IgG, diluted at a ratio of 1:5000. An ECL chemiluminescence kit (Solarbio, Beijing, China) was used to detect positive bands of PepN, PepO, SPD_1609 and trivalent vaccine in the different *S. pneumoniae* strains.

### 2.6 The cross-reactivity between antiserum and *S.pneumonia*e strains verified by ELISA

*S.pneumoniae* strains D39, 19F, 6B, CMCC203 and 2 clinical strains were inoculated on Columbia blood plates and cultured overnight at 37°C, 5%CO_2_. The following day the samples were obtained at Todd Hewitt Yeast (THY) at 28 - 29°C with gentle (100 *rpm*) shaking, and bacterial precipitation were obtained by centrifugation at 8,000 ×g for 15 min. Methods for obtaining total proteins were described in the literature (Jeon et al., 2023). Protein lysates were subsequently harvested by centrifugation at 15,000 ×g for 5 min. After quantification of BCA protein, the concentration was adjusted to 10 µg/mL, and the lysate (250 µL/well) was plated on a 96-well plates, then as described in section 2.4 ELISA. Mouse antiserum (each diluted 1:1000) against PepN, PepO, SPD_1609, PepO + PepN, PepO + SPD_1609, PepN + SPD_1609 and trivalent vaccine were used as primary antibodies, and the secondary antibody used was horseradish peroxidase (HRP)-conjugated goat anti-mouse IgG, diluted at a ratio of 1:5000 were added and incubated for 1 h at 37°C. The reaction was terminated by adding stop solution (Solarbio, Beijing, China) after 30 min of incubation with 3,3 ‘,5,5 ‘-tetramethylbenzidine (TMB) in the dark. Finally, absorbance was measured at 450 _nm._

### 2.7 Flow cytometry determination of CD4^+^ and CD8^+^ T cells

Peripheral blood lymphocyte populations in mice after immunization were examined as previously described (Hop et al., 2018). On day 7 after the last immunization of mice, 100 µL blood was collected from each mouse. Five microliters of fluorescein isothiocyanate (FITC)-conjugated rat anti-mouse CD4^+^ monoclonal antibody and 2 µL of phycoerythrin-conjugated rat anti-mouse CD8^+^ monoclonal antibody (BD Biosciences, Milipitas, CA, USA) were added and incubated for 30 min at room temperature in the dark. Red blood cell lysis buffer (1 mL) was added to each tube and the tubes were incubated for 10 min at room temperature until the red blood cells were completely lysed. The reaction was terminated by adding PBS (2 mL). Following centrifugation at 500 g for 5 minutes at 4°C, the pelleted cells were washed twice with PBS and then resuspended in 0.5 mL of PBS. Flow cytometry analysis was performed using a FACS Calibur flow cytometer (BD Biosciences).

### 2.8 Splenic cytokine ELISA

On day 14 after the last booster immunization, three mice were randomly selected from each group, and their spleens were aseptically isolated. Each spleen was ground using a tissue homogenizer and filtered through a 70 µm cell filter. Red blood cells were lysed using red blood cell lysate (Solarbio Science, Beijing, China) until the cell precipitate was milky-white. The resulting cell precipitate was washed three times with RPMI 1640 complete medium (HyClone, Logan, UT, USA). Splenocytes were seeded at 5 × 10^6^ cells/well in RPMI 1640 complete medium in 24-well cell culture plates and treated with 10 µg of recombinant protein antigen with endotoxin removed in PBS (sterilized by passage through a 0.2 μm filter). Cells were stimulated with concanavalin A as a positive control. The plates were incubated at 37°C in an atmosphere of 5% CO_2_. After 72 h, the culture supernatant was collected and stored at −80°C. Cytokine levels were measured using an ELISA kit (Proteintech, Rosemont, IL, United States). In this experiment, three mice were selected and the experiment was repeated independently three times in parallel.

### 2.9 Adhesion inhibition experiment

The binding of antiserum to *S. pneumoniae* was verified by an immunofluorescence assay as previously described (Cui et al., 2022). Briefly, A549 type II epithelial lung cancer cells (ATCC, Manassas, VA, USA) were cultured in Dulbecco’s modified Eagle’s medium containing 10% fetal bovine serum to the third generation and aliquots were inoculated in wells of 24-well plates at a density of 3 × 10^5^ cells/well at 37°C and 5% CO_2_ overnight. Strain 19F was mixed 1:1 with different antiserum, incubated at room temperature for 30 min, added to the culture wells of adherent growing A549 cells, and incubated at 37°C for 1 h. Subsequently, DiL cell membrane probe (Maokang Bio, Shanghai, China) and FITC-labeled sheep anti-mouse IgG (Beyotime Biotechnology, Shanghai, China) were added and incubated at 37°C for 45 min. Images were captured using an inverted fluorescence microscope DMi8 (Leica, Wetzlar, Germany) and quantitatively analyzed using Image J software (NIH, Bethesda, MD, USA). Only antiserum derived from the trivalent vaccine group was co-cultured with A549 cells as a negative control. For cell adhesion inhibition assays using the plate counting, A549 cells were co-cultured with the antiserum and *S. pneumoniae* mixture as described above. After 1 h of incubation, the plates were washed three times with sterile PBS. The cells were digested using trypsin (Thermo Fisher Scientific, Shanghai, China). Then the samples were diluted in a concentration gradient to inoculate on Blood Agar Plate (BAP) and incubate overnight at 37°C in the presence of 5% CO_2_ for bacterial counting.

### 2.10 RNA isolation and real-time quantitative reverse transcription polymerase chain reaction experiment

Total RNA was isolated from the lung tissues of mice in each group using Trizol reagent (Invitrogen, Carlsbad, CA, USA) following the manufacturer’s protocol. The quality and concentration of the extracted RNA were measured spectrophotometrically. Reverse transcription was then carried out to synthesize complementary DNA (cDNA) using the PrimeScript™ RT kit (TaKaRa Bio, Shiga, Japan) which includes a genomic DNA eraser. QRT-PCR was performed using TB Green Premix Ex Taq II (TaKaRa Bio) as previously described (Huang et al., 2019). β-actin served as the reference gene. The primer sequences for the cytokines of detection are provided in **Supplementary Table S1**.

### 2.11 Measurement of cytokine levels

On day 7 after the last immunization, each immunized mouse was intranasally colonized with *S. pneumoniae* 19F (1.0 × 10^6^ CFU/mL). Three days later, the mice were killed by cervical dislocation and nasal flush fluid was collected. The collected fluid was centrifuged at 1200 ×g for 10 min, and the supernatant was stored at –80°C. The expression levels of cytokines interleukin (IL)-1β, IL-10, interferon-gamma (IFN-γ), and tumor necrosis factor-alpha (TNF-α) in the supernatant were measured by ELISA kits (Aifang Bio, Henan, China).

### 2.12 Intranasal challenge experiment

On day 14 after the last immunization, mice in each group (n = 12) were anesthetized by inhaled isoflurane until coma, and then challenged with *S. pneumonia*e (serotype 19F) suspension (1.0 × 10^6^ CFU/30 µL) via nasal cavity. For the intranasal colonization experiment, mice in each group (n = 6) were randomly selected after 2-day infection. Firstly, the mice were fixed upside down, the trachea was separated, and the trachea was slowly injected with 300 µL sterile PBS, and the nasal cavity was rinsed twice, and all lavage fluid was collected. Finally, the nasal lavage solution, homogenized lung tissue, and homogenized brain tissue samples were diluted with aseptic PBS. Three plates were inoculated with each dilution for bacterial count, and the average number of colonies in the three plates was calculated as the final colony number. After overnight culture at 37°C and a 5% CO_2_ atmosphere, colony counting was performed to detect bacterial load. For the survival experiment of mice after infection, the remaining six mice from each group were used to monitor 7-day survival rate.

### 2.13 Histopathology

Histopathological analysis of lung tissues from infected mice was performed. The left lung lobes were fixed in 10% neutral buffered formalin (30 mL), processed for paraffin embedding, and sectioned. The sections were then mounted on slides and stained with hematoxylin and eosin to assess inflammatory changes in the lung tissue. A pathologist, who was blinded to the experimental group assignments, evaluated and scored the extent of inflammation in the lung tissue comprehensively based on five parameters as follows: (A) peribronchial/peribronchiolar inflammatory infiltrateaffectation; (B) peribronchial/peribronchiolar inflammatory infiltrateintensity; (C) bronchial/bronchiolar luminal exudate intensity; (D) perivascular inflammatory infiltrate affectation; (E) interstitial pneumonia intensity. According to previous studies (Mazzolini et al., 2023), the formula A + 3 (B + C + D) + E was used to calculate the final score (0–26 points) of mice in each group (n = 6).

### 2.14 Antiserum passive protection experiment

Female specific-pathogen-free BALB/c mice were randomly assigned to the following groups: PepN, PepO, SPD_1609, PepN + SPD_1609, PepO + SPD_1609, PepO + PepN, PepO + PepN + SPD_1609 (Trivalent), PBS negative control and PPV23 positive control (n=8 per group). On day 14 following the final immunization, 200 µL of cardiac blood was collected and administered via intraperitoneal injection to randomly assigned non-immunized mice. Mice were intraperitoneally injected with a lethal dose of *S. pneumoniae* D39 (600 CFU/100 µL) 1 hour after the cardiac serum was transferred to the mice. Tail venous blood was collected 48 h after infection, diluted in a gradient of sterile PBS, and coated onto BAP. After overnight culture at 37°C in a 5% CO_2_ atmosphere, colony counting was performed to detect bacterial load. The experiment was repeated three times in parallel. The 7-day survival rate of the mice was observed.

### 2.15 Statistical analysis

Statistical analysis for this study was conducted using SPSS version 29.0 software (IBM Corp., Armonk, NY, USA, Version 29.0). All datasets underwent normality and homogeneity of variance tests. For comparisons between two independent groups, we utilized the student’s t-test, unpaired t-test with Welch’s and Mann-Whitney U test. Multiple group comparisons were performed using one-way ANOVA, one-way ANOVA (Welch’s), Fisher’s exact test, and Kruskal-Wallis test. *Post hoc* analyses for multiple comparisons included Bonferroni correction, least significant difference (LSD) test, and Dunn’s test. Survival analysis was carried out using the log-rank (Mantel-Cox) test. Statistical significance was set at a *p*-value threshold of < 0.05.

## 3 Results

### 3.1 Preparation of antiserum and humoral immunization responses

**Figure 1:**
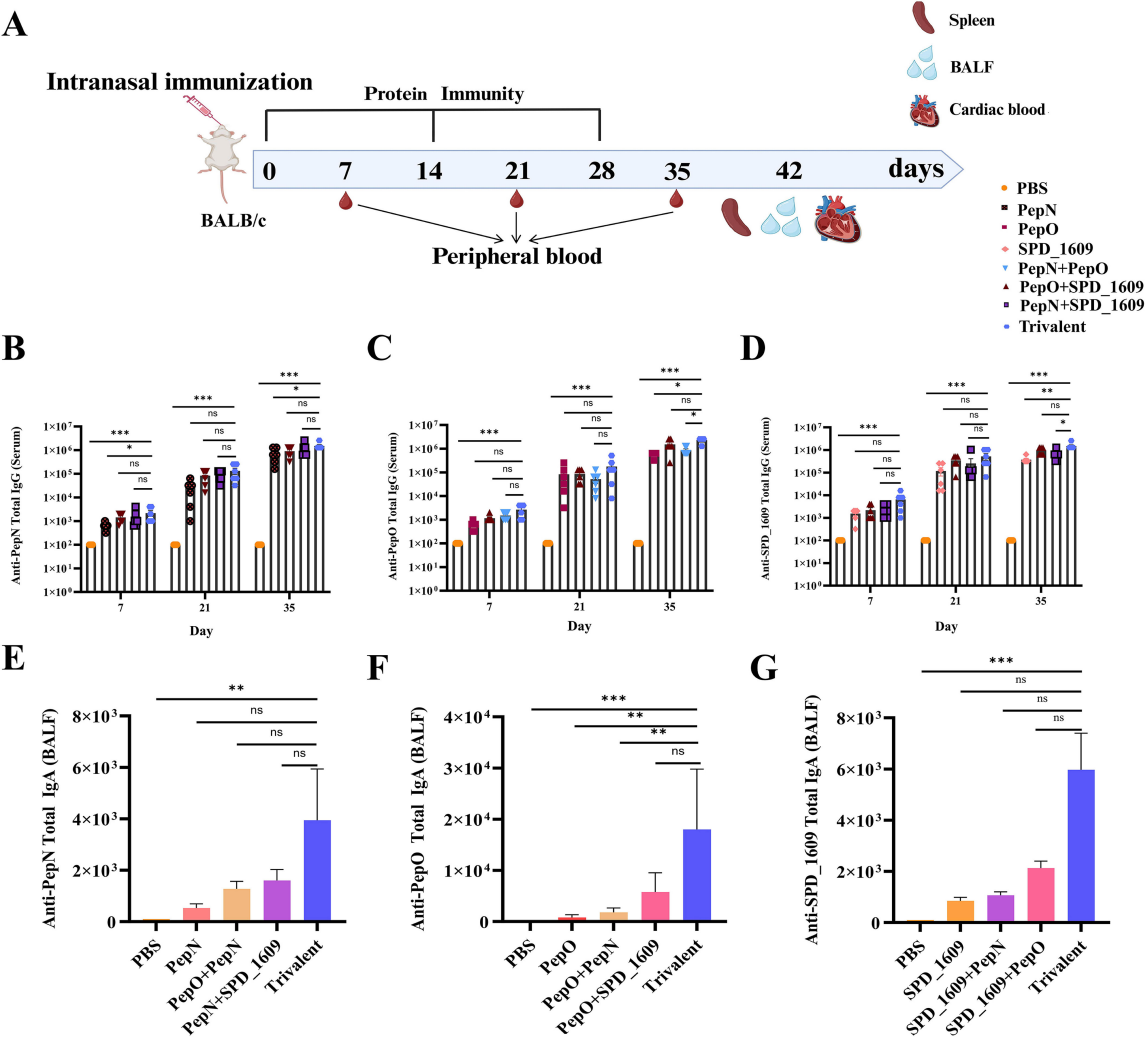
IgG and IgA antibodies elicited by trivalent component protein. **(A)** Immunization schedule and sample collection in BALB/c mice (n = 8 per group). **(B-D)** On days 7, 21, and 35 after immunization, the serum titers of anti-PepN, anti-PepO, and anti-SPD_1609 IgG antibodies in each group were determined using ELISA. **(E-G)** IgA antibody titers measured by ELISA in BALF collected on day 42 after immunization. Data are expressed as mean with 95% CI (n = 6), compared to the trivalent vaccine group (Bonferroni multiple comparisons test). ns, non-significant, *P* > 0.05, ^*^P < 0.05, ^**^P < 0.01, ^***^P < 0.001.

BALB/c mouse immunization was performed as illustrated in **Figure 1A**. Briefly, after the initial immunization of mice, sera were collected on days 7, 21, and 35. BALF was collected on day 42 to detect antibody titers. Overall, as immunization progressed, the IgG titers produced by all vaccine immunization groups gradually increased. Compared to the monovalent or bivalent vaccine group, the trivalent vaccine group tended to elicit a stronger IgG antibody response, especially after the third immunization. The IgG antibody titer in the trivalent vaccine group was >1.00 × 10^6^ (**Figures 1B–D**). In the BALF, all vaccine immunization groups were able to induce IgA antibody titers (**Figures 1E–G**), and the trivalent vaccine group showed significantly higher titers than the monovalent or bivalent vaccine group. These results suggested that the trivalent vaccine could significantly induce a humoral immunity (IgG) and mucosal immunity (IgA) response.

### 3.2 Evaluation of antiserum cross-reactivity against diverse bacterial strains (ELISA)

**Figure 2:**
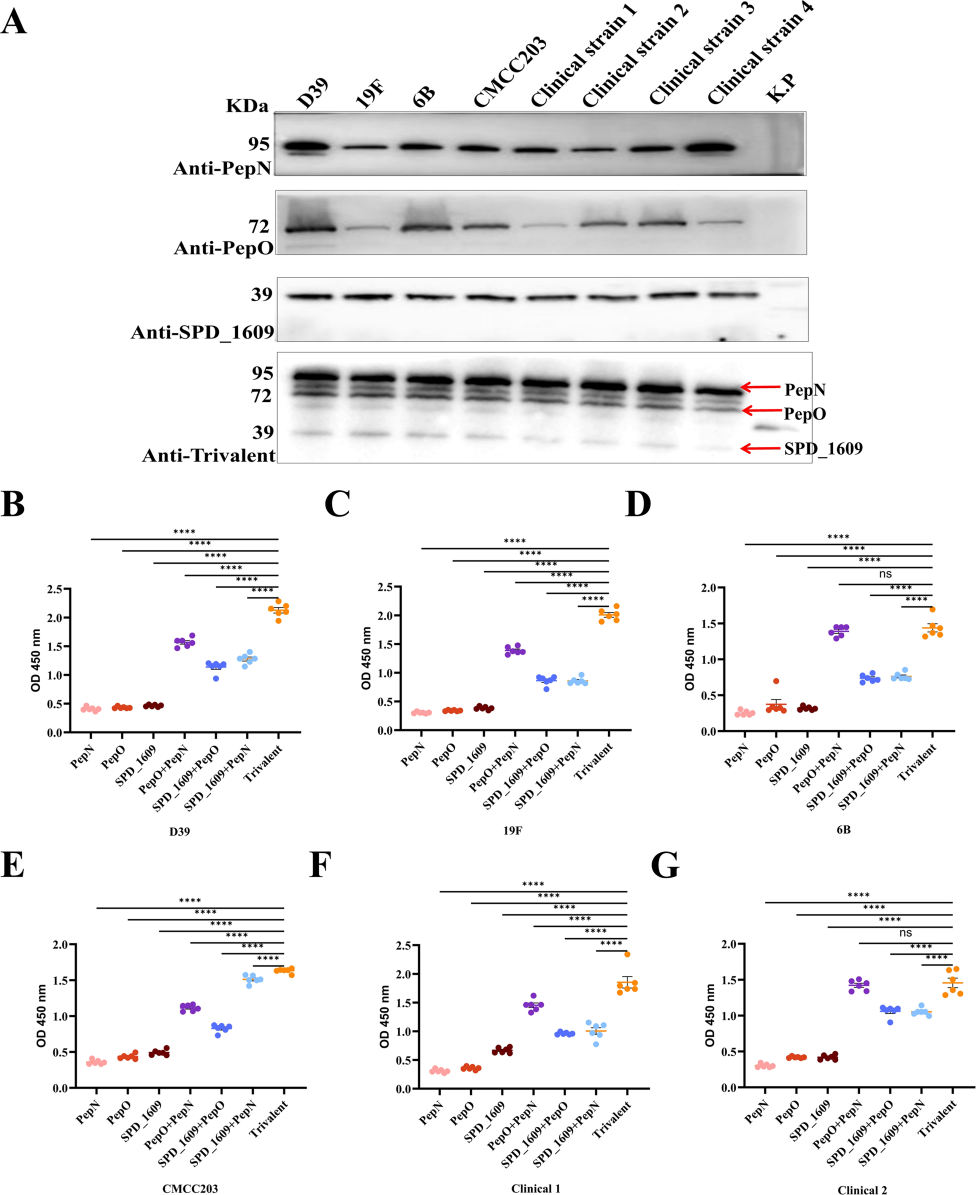
Evaluating the antigen-binding specificity and epitope recognition capacity of immune serum. **(A)** Western Blot analysis was employed to evaluate the antigen-binding specificity of post-immunization antisera generated by three monovalent protein-based vaccines: PepO, PepN, SPD_1609 and the trivalent. Lane 1–9 were D39, 19F, 6B, and CMCC203 (serotype 3), and four clinical strains and negative control *Klebsiella pneumoniae(K.p)*, respectively. **(B-G)** Detection of PepN, PepO and SPD_1609 in protein lysates of several *S. pneumoniae* strains. Total proteins were extracted from six strains of *S. pneumoniae*, including D39, 19F, 6B, CMCC203 (serotype 3), and two clinical strains, and coated on 96-well plates for ELISA. Anti-PepN or PepO or SPD_1609, anti-PepN + PepO or PepO + SPD_1609 or PepN + SPD_1609 and anti-trivalent IgG was used to detect PepN, PepO, and SPD_1609 in lysates, compared to anti-trivalent IgG (Dunn’s multiple comparisons test). ns, non-significant, *P* > 0.05, ^*^P < 0.05, ^**^P < 0.01, ^***^P < 0.001, ****P < 0.001.

To evaluate the conservation of these three antigens, we compared gene sequence similarity among 100 *S. pneumoniae* strains from the GenBank database. The analysis results revealed sequence identities of 99.76% for *pepn*, 99.74% for *pepo*, and 95.88% for *spd_1609* (**Supplementary Tables S1**-**S3**). Western blot analysis demonstrated that monovalent antisera specifically recognized their corresponding antigens in eight *S. pneumoniae* strains, while antiserum derived from the trivalent vaccine group concurrently detected all three antigens (**Figure 2A**). Furthermore, ELISA experiments with bacterial lysates revealed binding efficacy hierarchy: trivalent > bivalent > monovalent antisera when interacting with lysates from six *S. pneumoniae* strains (**Figures 2B–G**). Collectively, these findings confirm the superior binding efficiency of antiserum derived from the trivalent vaccine group to natively expressed pneumococcal antigens, compared to monovalent and bivalent antiserum.

### 3.3 Vaccine-induced cellular immune responses

**Figure 3:**
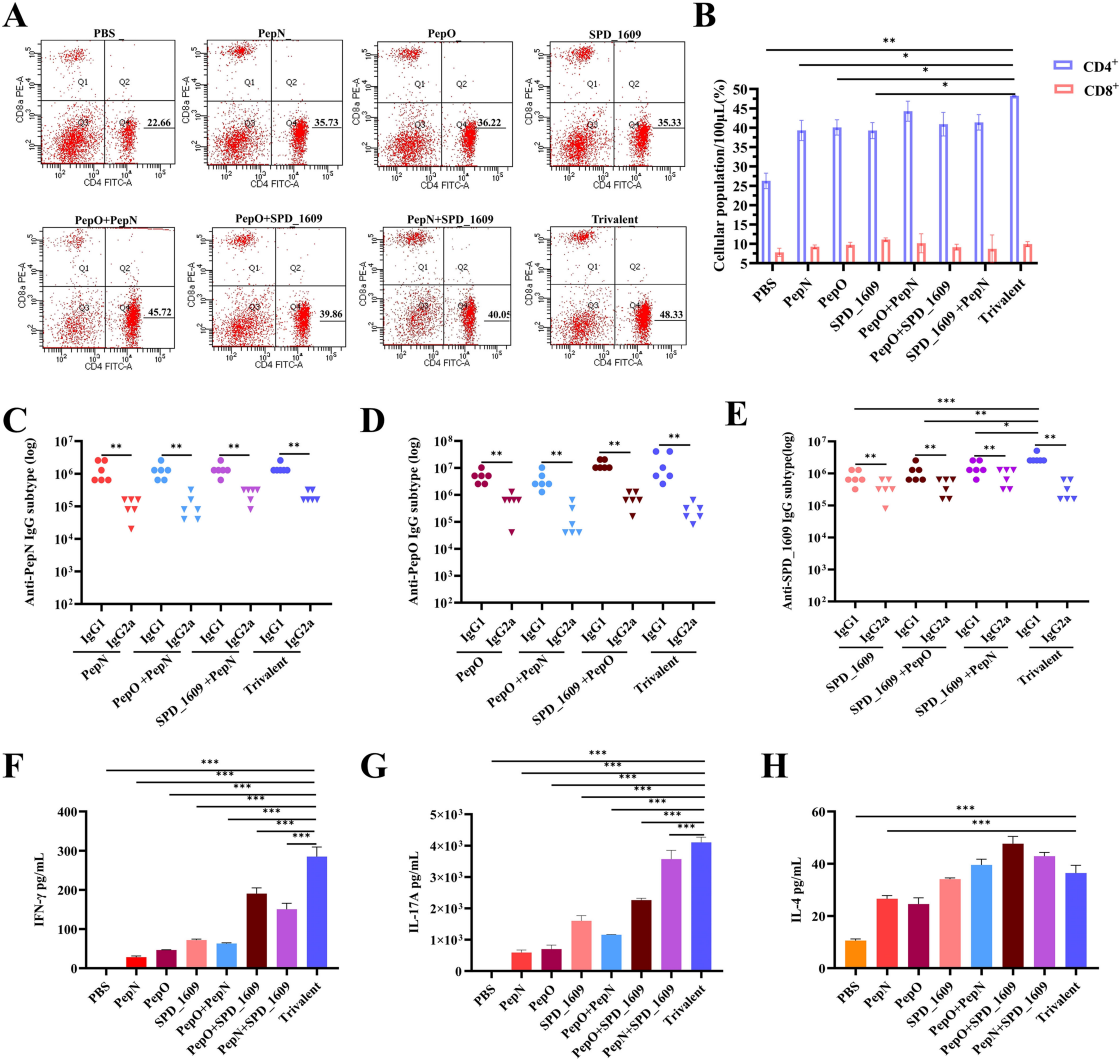
Analysis of the type of cellular immunity activated by vaccine immunization. CD4^+^ T cells and CD8^+^ T cells production by peripheral blood following mouse immunization. Flow cytometry images with CD4^+^ T cells and CD8^+^ T cells gated are shown **(A)**, and a summary of T lymphocyte subsets at day 42 after the first immunization are shown **(B)**, compared to the trivalent vaccine group (n = 3 per group) (Student’s t-test). **(C-E)** Analysis of serum antigen-specific IgG1 and IgG2a titers were analyzed on day 35 after the first immunization (n = 6) compared to the trivalent vaccine group (Dunn’s multiple comparisons test), and comparison of IgG1 with IgG2a using Mann-Whitney test. **(F-H)** On day 42 after the first immunization, splenocytes (5 × 10^6^ cells/well) were harvested from immunized mice (n = 3 per group) and co-cultured with 10 µg of the protein antigens at 37°C for 72 h. The supernatants of the cultures were analyzed for IFN-γ, IL-17A, and IL-4 expression levels using ELISA. Data are expressed as mean ± SEM, compared to the trivalent vaccine group (Dunn’s multiple comparisons test). ns, non-significant, *P* > 0.05, ^*^P < 0.05, ^**^P < 0.01, ^***^P < 0.001.

In natural infections, the activation of CD4^+^ and CD8^+^ T lymphocyte subsets are important in the clearance of pathogens, and T cell-dependent immune memory produced by CD4^+^ T cells prevents pneumococcal colonization of the nasopharynx (Al-Mariri et al., 2001; Malley et al., 2005). On day 42 after the first immunization, flow cytometry detection of T cell subsets in the peripheral blood showed that the ratio of lymphocytes produced by all vaccine immunization groups was CD4^+^ T cells > CD8^+^ T cells, with a higher number of CD4^+^ T peripheral blood lymphocytes in the trivalent vaccine group than the monovalent or bivalent vaccine group (**Figures 3A, B**). Additionally, detection of IgG1 suggests activation of the helper T cell 2 (Th2) cell response, and IgG2a suggests activation of the helper T cell 1 (Th1) cell response. On day 35 after the first immunization, high titers of IgG isotype antibody responses were observed in all vaccine groups, with IgG1 > IgG2a (**Figures 3C–E**). These results suggested the induction of a Th2-type biased immune response in all vaccine immunization groups. On day 42 after the first immunization, the protein antigens of each group were co-cultured with mouse spleen cells isolated after immunization with each group of vaccines for three days, and the cytokine levels in the culture supernatants were detected using ELISA kit. The result demonstrated that the trivalent vaccine group showed markedly elevated expression levels of IFN-γ (Th1) and IL-17A (Th17), compared to monovalent or bivalent vaccine group. All protein vaccine groups significantly induced IL-4 production compared with PBS group, except for PepN group, there was no significant difference between trivalent vaccine and monovalent or bivalent vaccine group (*P*>0.05) (**Figures 3F–H**). These finding suggested that the trivalent vaccine group induce cellular immune responses in Th1, Th2, and Th17.

### 3.4 Evaluation of antiserum binding efficiency to *S.pneumoniae*

**Figure 4:**
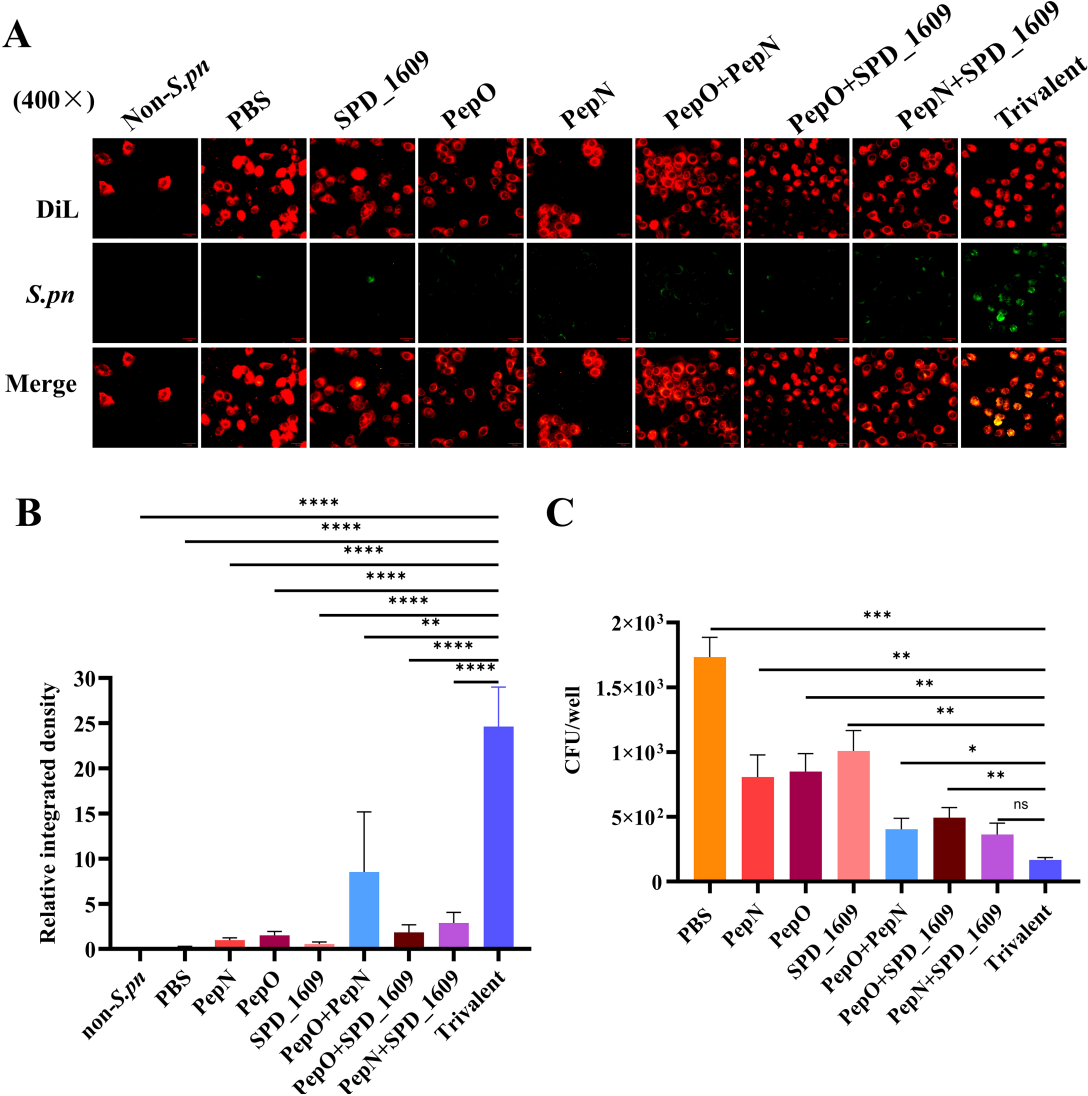
Assessment of antiserum interaction with *S. pneumoniae* and inhibitory capacity of *S. pneumoniae* adhesion to A549 cells. **(A)** Immunofluorescence imaging results of antiserum interaction with *S. pneumoniae*. DiL: labeling A549 cell membranes (red fluorescence); green fluorescence: indicating *S. pneumoniae* binding to antiserum; merge: synthetic image for the imaging results at different wavelengths, showing that *S. pneumoniae* successfully adhered to A549 cells. **(B)** Based on FITC green fluorescence signals, we performed quantitative analysis of fluorescence images using Image J software (Student’s t-test). **(C)** Analysis of the inhibitory capacity of antiserum against *S. pneumoniae* adhering to A549 cells, compared to the trivalent vaccine group (Dunn’s multiple comparisons test). Data are expressed as mean ± SEM. ns, non-significant, *P* > 0.05, ^*^P < 0.05, ^**^P < 0.01, ^***^P < 0.001, ****P < 0.001.

To evaluate the binding capacity of the antiserum to the surface of *S. pneumoniae*, we employed *S. pneumoniae* strain 19F as the antigen and lung cancer epithelial cells (A549) as the adhesion carrier for *S. pneumoniae*. Additionally, antiserum derived from each vaccine-immunized group, along with FITC-labeled goat anti-mouse IgG antibody, were used to ascertain the interaction between antiserum and *S. pneumoniae*. The fluorescence intensity of the antiserum derived from the trivalent vaccine group was significantly higher than those in the monovalent or bivalent vaccine group (**Figure 4A**). This result was corroborated by quantitative Image J fluorescence analysis (**Figure 4B**). The adhesion of pneumococci to host epithelial cells is a key link in *S. pneumoniae* infection (Hu et al., 2021), we evaluated the ability of different antiserum to inhibit the adhesion of *S. pneumoniae* to A549 cells. The results of the antibody adhesion inhibition experiment showed that the antiserum derived from the trivalent vaccine group significantly inhibited the adhesion of *S. pneumoniae* to A549 cells compared to the monovalent or bivalent vaccine group (*P* < 0.05, **Figure 4C**).

### 3.5 Impact of vaccination on *S. pneumoniae* colonization

**Figure 5:**
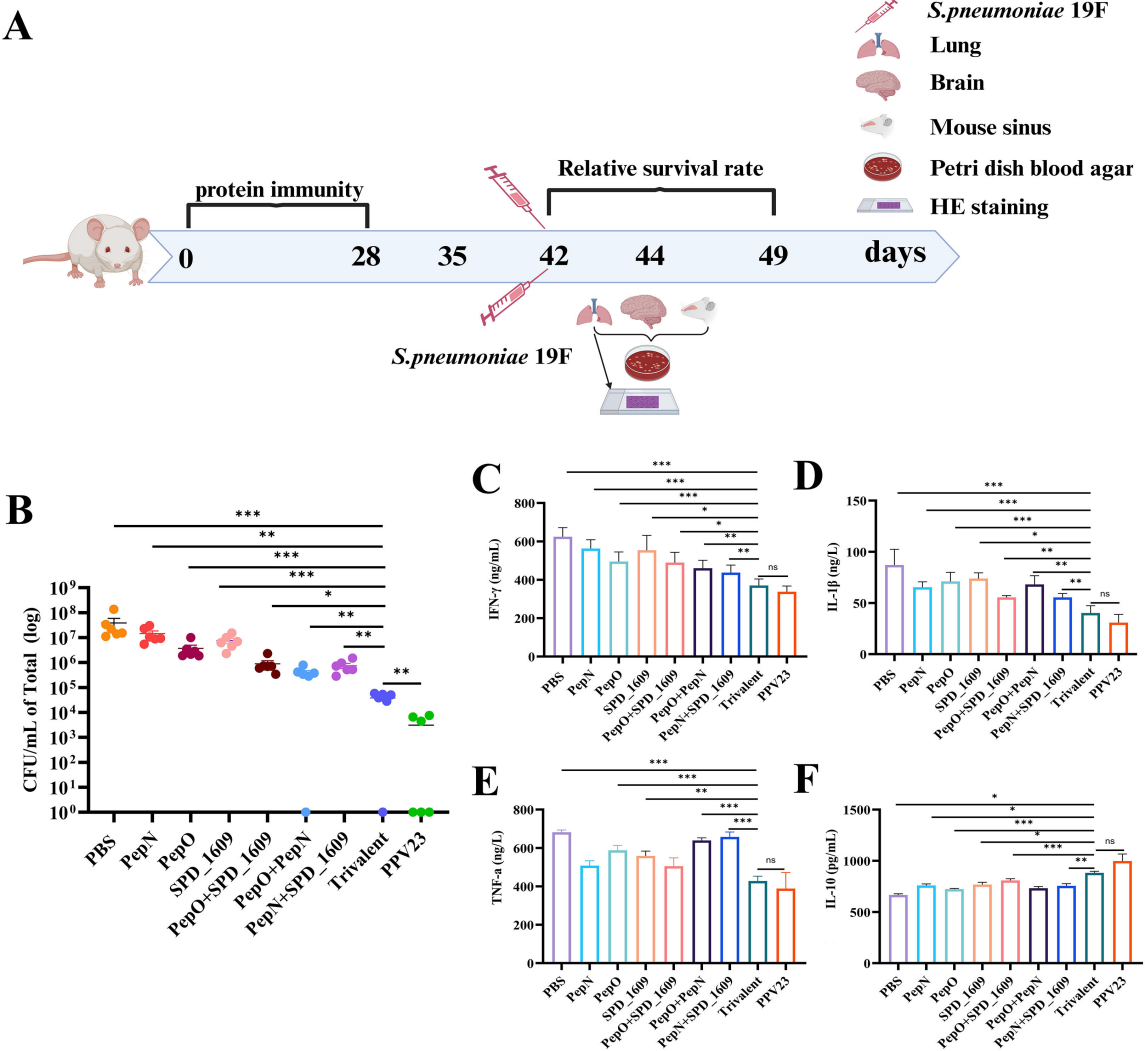
Bacterial load and cytokine expression levels after nasal infection with *S. pneumoniae* 19F in mice. **(A)** Timeline of intranasal colonization and collection of nasal lavage fluid and lung and brain tissue in mice. **(B)** Mice immunized with different vaccine groups were challenged with a sublethal dose (1 × 10^6^ CFU/mL) of 19F on day 14 after the last immunization and bacterial load was monitored (n = 6), compared to the trivalent vaccine group (Bonferroni multiple comparisons test). **(C-F)** Expression levels of inflammatory cytokines INF-γ **(C)**, IL-1β **(D)**, TNF-a **(E)**, and IL-10 **(F)** in the nasal fluids following vaccine immunization (n = 6). Data are expressed as mean ± SEM or 95% CI, compared to the trivalent vaccine group (Dunn’s multiple or Bonferroni multiple comparisons test). ns, non-significant, *P* > 0.05, ^*^P < 0.05, ^**^P < 0.01, ^***^P < 0.001.

Colonization of the nasopharynx by *S. pneumoniae* is a prerequisite for the invasion of the lungs or bloodstream. Inflammatory infections caused by *S. pneumoniae* colonizing the nasopharynx can spread to other tissues (Puchta et al., 2014; Khan et al., 2017). Accordingly, we constructed a colonization model of *S. pneumoniae* 19F strain to evaluate the potential protective effect of vaccination on preventing *S. pneumoniae* nasopharyngeal colonization. On day 14 after the last immunization, the mice were infected intranasally with *S. pneumoniae* 19F strain. On day 2 post-infection, the bacterial load in the nasal lavage fluid was determined using the dilution plate coating method (**Figure 5A**). Bacterial load was significantly reduced in the trivalent vaccine group compared to the monovalent or bivalent vaccine group (*P* < 0.05). The bacterial load of the trivalent vaccine group was higher than that of the PPV23 vaccine group (*P* < 0.01) (**Figure 5B**). Following infection, the body activates inflammatory regulation and antimicrobial responses by stimulating the secretion of relevant cytokines. To further investigate the potential role of cytokine regulation in maintaining immune system homeostasis, we analyzed cytokine expression levels in nasal lavage fluid. The results of ELISA revealed that the trivalent vaccine group reduced pro-inflammatory cytokines (IFN-γ, IL-1β, TNF-α) and elevated anti-inflammatory IL-10, compared to monovalent or bivalent vaccine groups (*P* < 0.05) (**Figures 5C–F**). According to the above results, it demonstrated that the trivalent vaccine exhibits superior efficacy in combating *S. pneumoniae* strain 19F colonization, compared to the monovalent or bivalent vaccine group.

### 3.6 Ameliorative effect and protective efficacy of vaccines against pneumococcal lung inflammation

**Figure 6:**
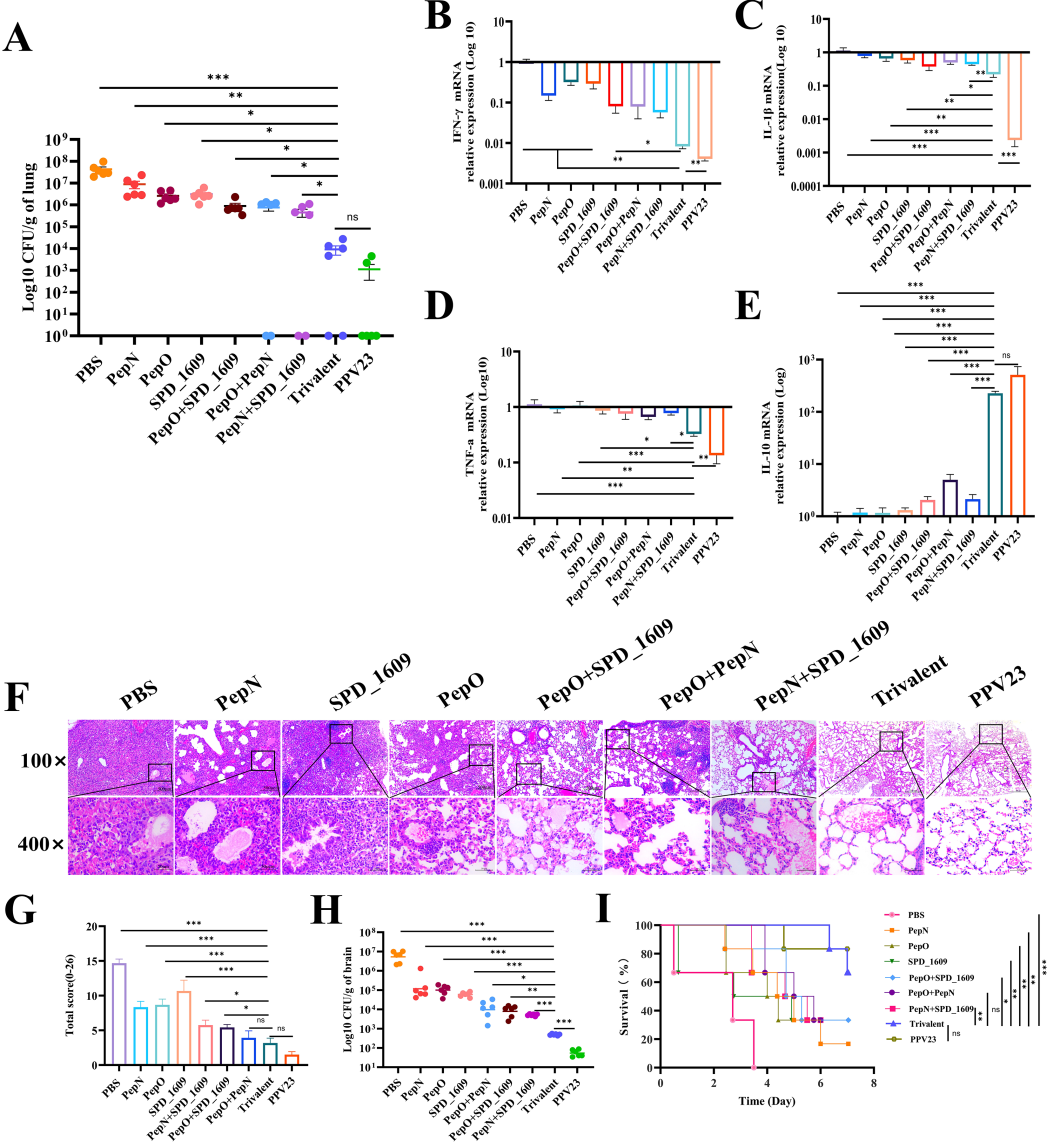
Evaluation of the protective effect of vaccines against pneumococcal pneumonia. **(A)** The bacterial load of *S. pneumoniae* detected at 48 h post-infection in lung (n = 6) **(B-E)** Expression levels of inflammatory cytokines INF-γ **(B)**, IL-1β **(C)**, TNF-a **(D)**, and IL-10 **(E)** in the lung compared to the trivalent vaccine group (Bonferroni multiple comparisons tests). **(F)** Hematoxylin and eosin staining of lung sections 2 days after infection (magnification 100× and 400×). **(G)** Lung pathology score at 2 days post-infection, compared to the trivalent vaccine group (LSD multiple comparisons test). **(H)** Bacterial load analysis of brain tissue homogenates was performed 2 days post-infection following nasopharyngeal colonization (n = 6), compared to the trivalent vaccine group (unpaired t-test with Welch’s or Bonferroni multiple comparisons tests). **(I)** The survival of each vaccine immunization group was recorded on day 7 in the nasopharyngeal colonization infection model (n = 6), compared to the trivalent vaccine group (log-rank Mantel-Cox test). Data are expressed as mean ± SEM or 95% CI. ns: non-significant, *P* > 0.05, ^*^P < 0.05, ^**^P < 0.01, ^***^P < 0.001.

Following intranasal infection, *S. pneumoniae* 19F can invade the lung tissue through the lower respiratory tract to cause pneumonia (Bhowmick et al., 2013). We examined the bacterial load, histopathological characteristics and changes in inflammatory factors in the lung tissues after infection to assess the ameliorative effect of the vaccine on lung inflammation. The bacterial load of homogenized lung tissue was significantly lower (*P* < 0.05) in the trivalent vaccine group than the monovalent or bivalent vaccine group, but there was no significant difference in bacterial load between the trivalent vaccine group and the PPV23 vaccine group (**Figure 6A**). The regulation of inflammatory factors is particularly important for the clearance of *S. pneumoniae*. The mRNA expression levels of IFN-γ, IL-1β, TNF-α and IL-10 in the lung tissues were analyzed by qRT-PCR. The results showed that the expression levels of pro-inflammatory factors IFN-γ, IL-1β, TNF-α were markedly down-regulated (*P* < 0.05), and the expression level of the anti-inflammatory cytokine IL-10 was significantly up-regulated (*P* < 0.001) in the trivalent vaccine group (**Figures 6B–E**), compared to the monovalent or bivalent vaccine group. Furthermore, histopathological examinations of lung tissue samples revealed severe infiltration of inflammatory cells, thickening of alveolar walls, collapse of alveolar structures, and notable consolidation of pulmonary alveolar after PBS immunization. Other vaccine groups exhibited improved lung histopathology. In particular, the trivalent vaccine group demonstrated a notable reduction in inflammatory cells, an intact alveolar structure, and a reduction in the overall pathological scores (**Figures 6F, G**).

In addition, we performed bacterial load analysis on homogenized brain tissue to verify whether *S. pneumoniae* had spread to this tissue. The trivalent vaccine group had a significantly reduced bacterial load in the brain tissue compared to the monovalent or bivalent vaccine group (*P* < 0.05). The bacterial load of the trivalent vaccine group was higher than that of the PPV23 vaccine group (*P* < 0.001) (**Figure 6H**). This study systematically evaluated the protective efficacy of the vaccine by constructing a model of *S. pneumoniae* serotype 19F nasopharyngeal colonization. The survival rate at 7 days after infection showed that the PBS negative control and PPV23 vaccine-positive control group was 0% and 83%, respectively. The survival rates of both the monovalent and bivalent vaccine groups were < 35%. In contrast, the survival rate in the trivalent vaccine group was 66.6% (*P* < 0.05), which was lower than that of the PPV23 vaccine group (83%), but the difference was not statistically significant (**Figure 6I**). These results suggest that the trivalent vaccine group offers greater protection against *S. pneumoniae* 19F infection in mice, compared to the monovalent or bivalent vaccine group.

### 3.7 Passive protection of antiserum against lethal challenge with *S. pneumoniae*

**Figure 7:**
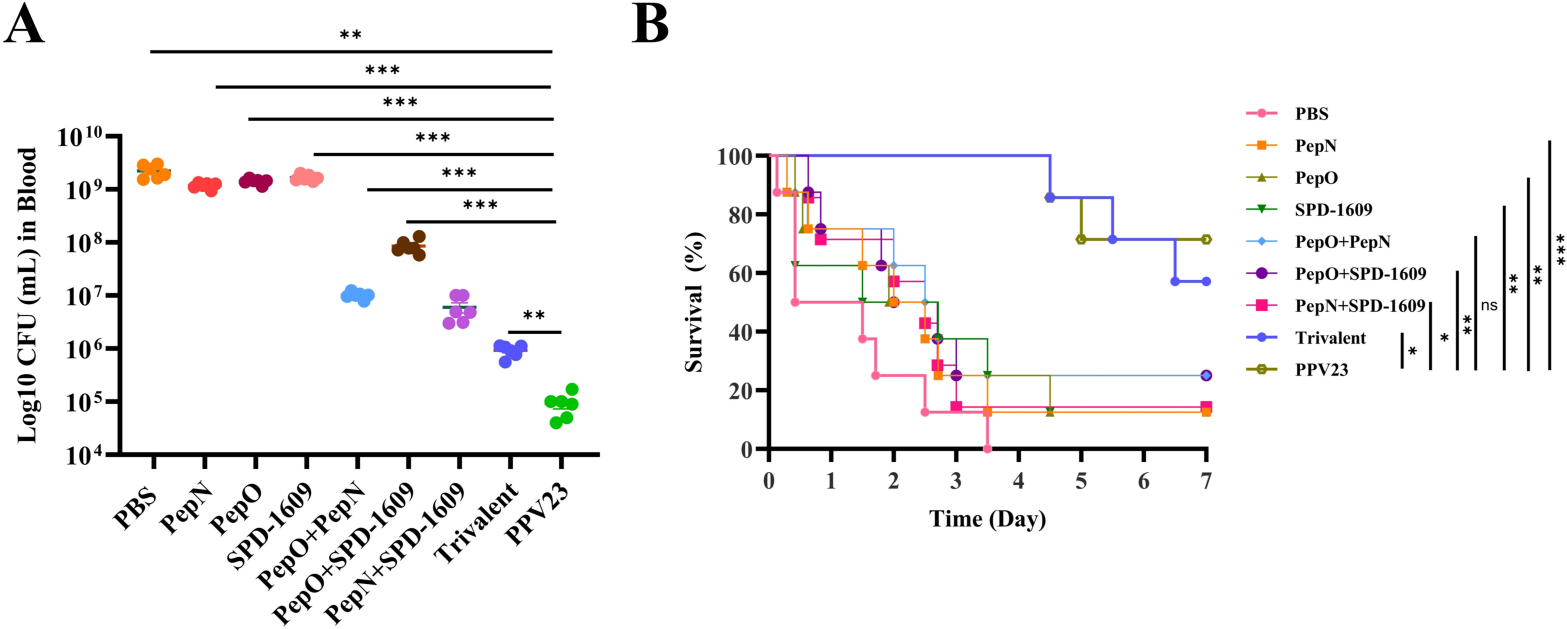
Bacterial load and survival rates after mice passively accepted antiserum. **(A)** Counting of bacterial load in peripheral blood collected from mice 2 days after infection was performed in a passive protection experiment involving the D39 strain and antiserum derived from each vaccine immunization group (n = 6), compared to the trivalent vaccine group (Dunn’s multiple comparisons test). **(B)** The 7-day survival rates of mice were monitored after the challenge (n = 7), compared to the trivalent vaccine group (log-rank Mantel-Cox test). Data are expressed as the mean ± SEM. ns, non-significant, *P* > 0.05, ^*^P < 0.05, ^**^P < 0.01, ^***^P < 0.001.

Humoral immunity is crucial for mice in resisting *S. pneumoniae* infection (Wang et al., 2022). Passive protection trials were performed to further confirm the efficacy of the antiserum. Antiserum derived from immunized mice were injected intraperitoneally to unimmunized mice simultaneously with a sublethal dose of *S. pneumoniae* strain D39. Serial dilution and plate counting of peripheral blood were performed, and the survival rate of mice was recorded. Antiserum derived from the trivalent vaccine group demonstrated a greater reduction in bacterial load in the peripheral blood than those from the monovalent or bivalent vaccine group (*P* < 0.01), The bacterial load of the trivalent vaccine group was higher than that of the PPV23 vaccine group (*P* < 0.01) (**Figure 7A**). Furthermore, the survival rate of the PBS negative control group and PPV23 vaccine-positive control group was 0% and 71.4%, respectively. The survival rate of mice receiving antiserum was < 25% in both the monovalent or bivalent vaccine group. In contrast, mice receiving antiserum derived from the trivalent vaccine group had a survival rate of 57.1% (**Figure 7B**). These results indicate that the antiserum derived from the trivalent vaccine group were partially protective against lethal strain challenge.

## 4 Discussion

Commercially available *S. pneumoniae* vaccines consist of different capsular serotypes, including the 23-valent *S. pneumoniae* polysaccharide vaccine (PPV-23) and the 7-, 10-, and 13-valent *S. pneumoniae* conjugate vaccines (PCV-7, PCV-10, and PCV-13, respectively). All these vaccines can only cover some serotypes of *S. pneumoniae*, thereby stimulating immune responses against specific serotypes of *S. pneumoniae* (Pichichero et al., 2016). Existing *S. pneumoniae* vaccines will not prevent infection with other serotypes of *S. pneumoniae*. Protein vaccines have been widely studied because they can induce non-serotype-dependent immunity, in particular, the combination of multiple proteins can trigger broader protective immunity in the host (Verhoeven et al., 2014; Chen et al., 2015; Scott et al., 2021; Cui et al., 2022). Some key *S. pneumoniae* virulence factors - including LytA, LytC, CbpD, PspC, PspA, Pht proteins, PiuA/PiaA, and CbpG - have emerged as promising vaccine candidates (Mahdi et al., 2008; Pichichero et al., 2016; Ali et al., 2020; Li et al., 2023b; Nahian et al., 2025). These proteins exhibit strong immunogenicity in animal models and can effectively block bacterial adhesion, biofilm formation, and invasive infection. Given the extensive antigenic variability of *S.pneumoniae*, monovalent vaccines are unlikely to provide satisfactory protection (Scott et al., 2021). To address this limitation, multivalent vaccine strategies targeting pathogens can provide broader bacterial strain coverage and enhanced protective efficacy. For example, the trivalent recombinant protein vaccine of *S. pneumoniae*, PlyD1, PhtD, and PcbA can protect young mice against lethal *S. pneumoniae* serotypes 6A and 3. In light of their safety and good immunogenicity in adults and young children, they have entered Phase II human clinical trials (Verhoeven et al., 2014; Brooks et al., 2015; Odutola et al., 2016; Xu et al., 2017).

Intranasal immunization is more effective in inducing mucosal and systemic immunity than intramuscular immunization, thereby stimulating a protective effect against *S. pneumoniae*. Intranasal immunization has many advantages, such as lower antigen dosage, safety, and non-invasiveness (Jeyanathan et al., 2018; Thakkar et al., 2018; Chiu et al., 2023) confirmed that intranasal administration of rlipo-PsaA, rPspAΔC, and rPspCΔC (LAAC) could stimulate the host to produce systemic mucosal immune response (IgA), thereby helping to protect against *S. pneumoniae* infection. Currently, screening and evaluating the protective effects of different protein combination vaccines against *S. pneumoniae* infection is the main direction of *S. pneumoniae* protein vaccine research. The present study investigated the protective effect and immune mechanism of intranasal immunization with a trivalent recombinant protein vaccine incorporating PepN, PepO, and SPD_1609 against *S. pneumoniae* infection. Our findings suggested that trivalent vaccine group elicit stronger immune responses and exhibit superior protective efficacy compared to monovalent or bivalent vaccine group.

An ideal protein vaccine is characterized by its capacity to induce high-titer specific antibodies and reduce bacterial colonization through antibody-dependent immunomodulatory mechanisms (Miao et al., 2023). In this study, the serum antibody titer test confirmed that the monovalent, bivalent, and trivalent vaccine immunization groups stimulated the host to produce high titers of IgG antibodies, and the trivalent vaccine immunization group displayed the stimulated production of a high titer of IgG antibodies in mice compared to the monovalent or bivalent vaccine immunization group. The homology analysis showed that the *pepn*, *pepo* and *spd_1609* genes had high homology among different serotypes of *S. pneumoniae.* Western blot and ELISA experiments also confirmed specific reactivity of antisera with target proteins across multiple *S. pneumoniae* serotypes. The binding ability of antibodies to *S. pneumoniae* is a key element in the immune defense mechanism of *S. pneumoniae* infection (Cui et al., 2022). Our results demonstrated that trivalent vaccine exhibited significantly higher green fluorescence intensity upon binding to *S. pneumoniae* surfaces compared with that from monovalent or bivalent vaccine group. These findings suggested that the antiserum derived from the trivalent vaccine group had superior binding efficiency to *S. pneumoniae.* The antibody adhesion inhibition assay also showed that the antiserum derived from the trivalent vaccine group were more effective at inhibiting the adhesion of *S. pneumoniae* to A549 epithelial cells. IgA antibody acts mainly at the mucosal surface, and it exists in the respiratory mucosa and other parts in the form of secretory IgA (sIgA) to bind to the antigen on the surface of *S. pneumoniae* and prevent the adhesion of bacteria to mucosal epithelial cells (Park et al., 2024). The present study confirmed that the three recombinant proteins PepN, PepO, and SPD_1609 (monovalent, bivalent, and trivalent vaccine groups) could induce the production of high titers of IgA type antibody in BALF in mice. Mice receiving the trivalent vaccine group produced high titers of IgA antibody in comparison to mice immunized with the monovalent or bivalent vaccine group. In the *S. pneumoniae* nasal challenge model, the trivalent vaccine group more effectively reduced the number of *S. pneumoniae* colonizing and infecting bacteria in nasopharyngeal and lung tissue.

The activation of CD4^+^ and CD8^+^ T lymphocyte subsets is important in the host clearance of pathogens, and T cell-dependent immune memory generated by CD4^+^ T cells can prevent *S. pneumoniae* colonization in the nasopharynx (Jansen et al., 2019). CD4^+^ T helper cells consist of Th1 and Th2 subtypes. Th1 CD4^+^ T cells mainly participate in the activation of CD8^+^ cytotoxic T cells and mediate cellular immunity. However, Th2 CD4^+^ T cells focus on activating humoral immune responses (Luo et al., 2006). Therefore, the specific type of immune response induced can be revealed by measuring the changes in the number of CD4^+^ and CD8^+^ T cells in the blood after immunization, especially the levels of key Th1-type and Th2-type cytokines. Notably, our results showed that immunization with trivalent vaccine could significantly increase the proportion of CD4^+^ T cells compared with monovalent or bivalent vaccine group. Furthermore, the analysis of antibody subtypes suggested that all immunization groups induced Th2-biased immune responses, which may be related to the use of CTB as an immune adjuvant. Studies have documented that CTB adjuvant administration elicits immune polarization toward Th2-type responses (Holmgren et al., 2003; Sun et al., 2022). In addition, cytokine detection results in spleen cells indicated that the trivalent vaccine group elicited higher Th1 (INF-γ) and Th17 (IL-17A) cellular immune responses, compared to the monovalent or bivalent vaccine group. However, the trivalent vaccine group exhibited lower levels of Th2 (IL-4) responses relative to the monovalent or bivalent vaccine group, which may be due to a feedback inhibitory effect between IL-4 (Th2) and IL-17A (Th17) (Mureithi et al., 2009). These results dedicated that all three recontaminated proteins elicited a polarized Th1/Th17 cellular immune response in CD4^+^ T cells, which is essential for protective immunity against *S. pneumoniae* lung infection.

The IL-10 anti-inflammatory cytokine has an important role in the evolution of bacterial pneumonia. The up-regulation of IL-10 expression significantly improves the clearance rate of *Pseudomonas aeruginosa* in lungs (Steinhauser et al., 1999) This improvement might be related to the inhibitory effect of IL-10 on pro-inflammatory cytokine (TNF-a) (Reddy et al., 2001; Nagata and Nishiyama, 2021). Latifi et al. performed relevant studies involving a mouse model of sepsis confirming that the deficiency of IL-10 can promote the significant increase of TNF-α and IL-6, leading to high mortality. Taken together, these findings indicate that IL-10 is necessary for the balanced regulation of host pro-inflammatory cytokine responses (Latifi et al., 2002; Ouyang et al., 2011). The results of cytokine detection in homogenized lung tissue in this study confirmed that the trivalent vaccine group antigen could stimulate a higher level of the IL-10 anti-inflammatory factor in the lung tissue of mice infected with *S. pneumoniae*, compared to the monovalent or bivalent vaccine group. Simultaneously, the levels of pro-inflammatory cytokines (IFN-γ, TNF-a, IL-1β) were significantly decreased. A similar phenomenon was observed in the nasal lavage fluid. In addition, lung pathological observations confirmed that the trivalent vaccine group had a clearer alveolar structure and no significant inflammatory cell infiltration, compared with the monovalent or bivalent vaccine group. These findings indicate that the equilibrium of the immune response regulation of inflammatory cytokines is essential for alleviating pulmonary inflammatory injury in mice immunized with a trivalent vaccine via the nasal route. Subsequent studies by our group will comprehensively address the protective effects and safety of trivalent vaccine immunization via different administration routes.

## 5 Conclusion

Immunization with the trivalent vaccine produced better immunogenicity and protective effects than the monovalent or bivalent vaccine-immunized group. The trivalent vaccine also can induce a strong humoral immune response, as well as Th1, Th2, and Th17 cellular immune responses. The antiserum derived from the trivalent vaccine group demonstrated significant binding affinity to *S. pneumoniae*, effectively inhibiting its adhesion to A549 epithelial cells when compared to antisera obtained from monovalent or bivalent vaccine group. Mice immunized with the trivalent vaccine displayed a significantly reduced number of colonizing bacteria in the nasopharynx after challenge infection with *S. pneumoniae* strain 19F. In addition, lung tissue injury and inflammation were lessened. In the challenge infection experiment, the survival rate of mice immunized with the trivalent vaccine significantly improved after nasal challenge. The collective results implicate the trivalent vaccine composed of PepN, PepO, and SPD_1609 as a promising candidate *S. pneumoniae* multivalent vaccine.

## Data Availability

The raw data supporting the conclusions of this article will be made available by the authors, without undue reservation.
