# Supplementary material for: Intranasal trivalent candidate vaccine elicits broad humoral and cellular immunity against pneumococcal pneumonia
Source: Front Cell Infect Microbiol. 2025 Jun 27;15:1563661. doi: 10.3389/fcimb.2025.1563661 (PMC12245901; doi:10.3389/fcimb.2025.1563661)
Supplement: Supplementary file 1 [file DataSheet1.pdf]

frontiers

Supplementary Material

Intranasal trivalent candidate vaccine elicits broad humoral and cellular immunity against pneumococcal pneumonia

Table S1 Conservativeness analysis of antigenic sequences (PepN) was found in NCBI to be present in the following 100 genomes, with 99.76-100% nucleic acid identity).

Description	Scientific Name	Max Score	Total Score	Query Cover	E value	Per. ident	Acc. Len	Accession
Streptococcus pneumoniae TIGR4 chromosome, complete genome	Streptococcus pneumoniae TIGR4	4704	4704	100%	0	100	2153837	CP155539.1
Streptococcus pneumoniae Xen35, complete genome	Streptococcus pneumoniae	4704	4704	100%	0	100	2168552	CP025256.1
Neisseria gonorrhoeae FA 1090, complete genome	Streptococcus pneumoniae	4704	4704	100%	0	100	2153840	CP035239.1
Neisseria gonorrhoeae strain WHO_B_2024 chromosome, complete genome	Streptococcus pneumoniae	4704	4704	100%	0	100	2139846	CP038252.1
Neisseria gonorrhoeae strain WHO_beta_2024 chromosome, complete genome	Streptococcus pneumoniae	4704	4704	100%	0	100	2180605	AP018936.1
Neisseria gonorrhoeae strain WHO_alpha_2024 chromosome, complete genome	Streptococcus pneumoniae TIGR4	4704	4704	100%	0	100	2160842	AE005672.3

Neisseria gonorrhoeae strain 1123850 chromosome, complete genome	Streptococcus pneumoniae TIGR4	4704	4704	100%	0	100	2153849	CP089948.1
Neisseria gonorrhoeae strain ATCC 49226 chromosome, complete genome	Streptococcus pneumoniae	4704	4704	100%	0	100	2243694	AP028611.1
Neisseria gonorrhoeae strain FQ82 chromosome, complete genome	Streptococcus pneumoniae	4693	4693	100%	0	99.92	2107778	LR536831.1
Neisseria gonorrhoeae strain FQ48 chromosome, complete genome	Streptococcus pneumoniae	4687	4687	100%	0	99.88	1995077	LR536839.1
Neisseria gonorrhoeae strain FQ04 chromosome, complete genome	Streptococcus pneumoniae	4687	4687	100%	0	99.88	2071654	CP038251.1
Neisseria gonorrhoeae strain FQ84 chromosome, complete genome	Streptococcus pneumoniae	4687	4687	100%	0	99.88	2150845	CP035235.1
Neisseria gonorrhoeae strain FDAARGOS_207 chromosome, complete genome	Streptococcus pneumoniae	4687	4687	100%	0	99.88	2122938	LR216069.1
Neisseria gonorrhoeae NG-k51.05 chromosome, complete genome	Streptococcus pneumoniae	4687	4687	100%	0	99.88	2108520	CP035234.1
Neisseria gonorrhoeae strain WHO_H_2024 chromosome, complete genome	Streptococcus pneumoniae	4687	4687	100%	0	99.88	2066045	AP028610.1
Neisseria gonorrhoeae strain NCTC13800 genome assembly, chromosome: 1	Streptococcus pneumoniae	4687	4687	100%	0	99.88	2086119	LR216063.1
Neisseria gonorrhoeae strain NCTC13798 genome assembly, chromosome: 1	Streptococcus pneumoniae	4687	4687	100%	0	99.88	2066045	AP028605.1
Neisseria gonorrhoeae strain RIVM0640, complete genome	Streptococcus pneumoniae	4687	4687	100%	0	99.88	2081497	LR216033.1
Neisseria gonorrhoeae strain 2004S05-027 chromosome, complete genome	Streptococcus pneumoniae	4687	4687	100%	0	99.88	2079937	AP026917.1

Neisseria gonorrhoeae strain 2010C02-038 chromosome, complete genome	Streptococcus pneumoniae	4687	4687	100%	0	99.88	2076043	CP137110.1
Neisseria gonorrhoeae strain 2016E02-233 chromosome, complete genome	Streptococcus pneumoniae	4687	4687	100%	0	99.88	2098679	CP035244.1
Neisseria gonorrhoeae isolate 632_2023 genome assembly, chromosome: 1	Streptococcus pneumoniae	4682	4682	100%	0	99.84	2105368	LR216032.1
Neisseria gonorrhoeae strain WHO_Y genome assembly, chromosome: 1	Streptococcus pneumoniae	4682	4682	100%	0	99.84	2066251	LR216064.1
Neisseria gonorrhoeae strain WHO_V genome assembly, chromosome: 1	Streptococcus pneumoniae	4682	4682	100%	0	99.84	2057144	CP025838.1
Neisseria gonorrhoeae strain WHO L genome assembly, chromosome: 1	Streptococcus pneumoniae	4682	4682	100%	0	99.84	2051693	CP139862.1
Neisseria gonorrhoeae strain 34530 chromosome, complete genome	Streptococcus pneumoniae	4682	4682	100%	0	99.84	2157682	LS483450.1
Neisseria gonorrhoeae strain 2022NG-0032 plasmid p2022NG-0032_1, complete sequence	Streptococcus pneumoniae	4682	4682	100%	0	99.84	2110175	CP137111.1
Neisseria gonorrhoeae strain FA6140, complete genome	Streptococcus pneumoniae	4682	4682	100%	0	99.84	2154542	LR216061.1
Neisseria gonorrhoeae strain 9112 chromosome, complete genome	Streptococcus pneumoniae	4682	4682	100%	0	99.84	2091173	AP026925.1
Neisseria gonorrhoeae strain 10272 chromosome, complete genome	Streptococcus pneumoniae	4682	4682	100%	0	99.84	2072679	CP137114.1
Neisseria gonorrhoeae strain 10231 chromosome, complete genome	Streptococcus pneumoniae	4682	4682	100%	0	99.84	2148358	AP026919.1
Neisseria gonorrhoeae strain 9071 chromosome, complete genome	Streptococcus pneumoniae	4682	4682	100%	0	99.84	2057685	AP026915.1
Neisseria gonorrhoeae strain 1137292 chromosome	Streptococcus pneumoniae	4682	4682	100%	0	99.84	2069030	CP169564.1
Neisseria gonorrhoeae strain 1130991 chromosome, complete genome	Streptococcus pneumoniae	4682	4682	100%	0	99.84	2108184	CP137102.1

Neisseria gonorrhoeae strain 1081168 chromosome, complete genome	Streptococcus pneumoniae	4682	4682	100%	0	99.84	2102932	LR216054.1
Neisseria gonorrhoeae strain 10239 chromosome, complete genome	Streptococcus pneumoniae	4682	4682	100%	0	99.84	2101669	LR216057.1
Neisseria gonorrhoeae strain 10538 chromosome, complete genome	Streptococcus pneumoniae	4682	4682	100%	0	99.84	2220570	CP168299.1
Neisseria gonorrhoeae strain 10269 chromosome, complete genome	Streptococcus pneumoniae	4682	4682	100%	0	99.84	2035403	AP026930.1
Neisseria gonorrhoeae strain 10704 chromosome, complete genome	Streptococcus pneumoniae	4682	4682	100%	0	99.84	2082222	LR216051.1
Neisseria gonorrhoeae strain 9464 chromosome, complete genome	Streptococcus pneumoniae	4680	4680	100%	0	99.84	2109408	CP139825.1
Neisseria gonorrhoeae strain 9399 chromosome, complete genome	Streptococcus pneumoniae	4676	4676	100%	0	99.8	2169584	CP031248.1
Neisseria gonorrhoeae strain 10529 chromosome, complete genome	Streptococcus pneumoniae	4676	4676	100%	0	99.8	2074160	LR536843.1
Neisseria gonorrhoeae strain 10727 chromosome, complete genome	Streptococcus pneumoniae	4676	4676	100%	0	99.8	2057382	LR216052.1
Neisseria gonorrhoeae strain 10500 chromosome, complete genome	Streptococcus pneumoniae	4676	4676	100%	0	99.8	2091587	AP026914.1
Neisseria gonorrhoeae strain 10562 chromosome, complete genome	Streptococcus pneumoniae	4676	4676	100%	0	99.8	2088058	LR216014.1
Neisseria gonorrhoeae strain 10702 chromosome, complete genome	Streptococcus pneumoniae	4676	4676	100%	0	99.8	2052251	LR216039.1
Neisseria gonorrhoeae strain 10574 chromosome, complete genome	Streptococcus pneumoniae	4676	4676	100%	0	99.8	2099956	CP139863.1
Neisseria gonorrhoeae strain 9431 chromosome, complete genome	Streptococcus pneumoniae	4676	4676	100%	0	99.8	2095224	CP155537.1
Neisseria gonorrhoeae strain 10562 chromosome, complete genome	Streptococcus pneumoniae	4676	4676	100%	0	99.8	2136743	CP113266.1

Neisseria gonorrhoeae strain 10819 chromosome, complete genome	Streptococcus pneumoniae	4676	4676	100%	0	99.8	2039791	CP155800.1
Neisseria gonorrhoeae strain 10743 chromosome, complete genome	Streptococcus pneumoniae	4676	4676	100%	0	99.8	2115408	LR134294.1
Neisseria gonorrhoeae strain 10744 chromosome, complete genome	Streptococcus pneumoniae	4676	4676	100%	0	99.8	2179100	CP137113.1
Neisseria gonorrhoeae strain 10525 chromosome, complete genome	Streptococcus pneumoniae	4676	4676	100%	0	99.8	2156586	AP026921.1
Neisseria gonorrhoeae strain 10537 chromosome, complete genome	Streptococcus pneumoniae	4676	4676	100%	0	99.8	2030186	CP079923.1
Neisseria gonorrhoeae strain 10536 chromosome, complete genome	Streptococcus pneumoniae	4676	4676	100%	0	99.8	2009151	CP102583.1
Neisseria gonorrhoeae strain 10268 chromosome, complete genome	Streptococcus pneumoniae	4676	4676	100%	0	99.8	2046572	CP027540.1
Neisseria gonorrhoeae strain 9126 chromosome, complete genome	Streptococcus pneumoniae D39	4676	4676	100%	0	99.8	2034212	CP118285.1
Neisseria gonorrhoeae strain 10720 chromosome, complete genome	Streptococcus pneumoniae	4676	4676	100%	0	99.8	2046551	CP061208.1
Neisseria gonorrhoeae strain 9343 chromosome, complete genome	Streptococcus pneumoniae	4676	4676	100%	0	99.8	2001615	CP035241.1
Neisseria gonorrhoeae strain 10577 chromosome, complete genome	Streptococcus pneumoniae	4676	4676	100%	0	99.8	2096290	CP173225.1
Neisseria gonorrhoeae strain 9458 chromosome, complete genome	Streptococcus pneumoniae	4676	4676	100%	0	99.8	2001396	LR216027.1
Neisseria gonorrhoeae strain 9460 chromosome, complete genome	Streptococcus pneumoniae	4676	4676	100%	0	99.8	2081720	CP139861.1
Neisseria gonorrhoeae strain 98D159 chromosome, complete genome	Streptococcus pneumoniae	4676	4676	100%	0	99.8	2088360	AP026927.1
Neisseria gonorrhoeae strain O2D156 chromosome, complete genome	Streptococcus pneumoniae	4676	4676	100%	0	99.8	2032511	CP118286.1

	D39							
Neisseria gonorrhoeae strain 3648 chromosome	Streptococcus pneumoniae	4676	4676	100%	0	99.8	2038121	CP019299.1
Neisseria gonorrhoeae strain WHO_A_2024 chromosome, complete genome	Streptococcus pneumoniae	4676	4676	100%	0	99.8	2109726	CP031246.1
Neisseria gonorrhoeae strain WHO_I_2024 chromosome, complete genome	Streptococcus pneumoniae	4676	4676	100%	0	99.8	2118406	AP028609.1
Neisseria gonorrhoeae strain WHO_J_2024 chromosome, complete genome	Streptococcus pneumoniae	4676	4676	100%	0	99.8	2054748	AP028601.1
Neisseria gonorrhoeae strain WHO_R_2024 chromosome, complete genome	Streptococcus pneumoniae	4676	4676	100%	0	99.8	2096231	CP031245.1
Neisseria gonorrhoeae strain 2018S05-288 chromosome, complete genome	Streptococcus pneumoniae	4676	4676	100%	0	99.8	2046573	LS483374.1
Neisseria gonorrhoeae strain 2020S05-258 chromosome, complete genome	Streptococcus pneumoniae	4676	4676	100%	0	99.8	2086667	CP035261.1
Neisseria gonorrhoeae strain 2020N07-265 chromosome, complete genome	Streptococcus pneumoniae	4676	4676	100%	0	99.8	2138920	CP137106.1
Neisseria gonorrhoeae isolate SGC-23-001 genome assembly, chromosome: contig1	Streptococcus pneumoniae	4676	4676	100%	0	99.8	2163419	CP137112.1
Neisseria gonorrhoeae strain NJ195417 chromosome, complete genome	Streptococcus pneumoniae	4676	4676	100%	0	99.8	2103319	LR216015.1
Neisseria gonorrhoeae strain NJ196610 chromosome, complete genome	Streptococcus pneumoniae	4676	4676	100%	0	99.8	2041494	CP036529.1
Neisseria gonorrhoeae strain NJ1914215 chromosome, complete genome	Streptococcus pneumoniae gamPNI0373	4676	4676	100%	0	99.8	2064154	CP001845.1
Neisseria gonorrhoeae strain NJ1911400	Streptococcus pneumoniae	4676	4676	100%	0	99.8	2078470	CP118288.1

chromosome, complete genome	D39							
Neisseria gonorrhoeae strain NJ1913940 chromosome, complete genome	Streptococcus pneumoniae D39	4676	4676	100%	0	99.8	2037657	CP118287.1
Neisseria gonorrhoeae strain NJ197542 chromosome, complete genome	Streptococcus pneumoniae	4676	4676	100%	0	99.8	2039982	LS483390.1
Neisseria gonorrhoeae strain NJ1914646 chromosome, complete genome	Streptococcus pneumoniae D39	4676	4676	100%	0	99.8	2046116	CP000410.2
Neisseria gonorrhoeae strain NJ208756 chromosome, complete genome	Streptococcus pneumoniae	4676	4676	100%	0	99.8	2227883	AP026916.1
Neisseria gonorrhoeae strain NJ208430 chromosome, complete genome	Streptococcus pneumoniae	4676	4676	100%	0	99.8	2147673	LR216040.1
Neisseria gonorrhoeae strain NJ209649 chromosome, complete genome	Streptococcus pneumoniae R6	4676	4676	100%	0	99.8	2038615	AE007317.1
Neisseria gonorrhoeae strain 1145734 chromosome, complete genome	Streptococcus pneumoniae	4676	4676	100%	0	99.8	2096423	AP018938.1
Neisseria gonorrhoeae strain 10588 chromosome, complete genome	Streptococcus pneumoniae	4676	4676	100%	0	99.8	2099632	AP026923.1
Neisseria gonorrhoeae strain 10612 chromosome, complete genome	Streptococcus pneumoniae	4676	4676	100%	0	99.8	2147252	OX244288.1
Neisseria gonorrhoeae strain H035 chromosome, complete genome	Streptococcus pneumoniae	4676	4676	100%	0	99.8	2038617	CP038808.1
Neisseria gonorrhoeae strain 10791 chromosome, complete genome	Streptococcus pneumoniae	4676	4676	100%	0	99.8	2046574	CP035238.1
Neisseria gonorrhoeae strain 10638 chromosome, complete genome	Streptococcus pneumoniae	4676	4676	100%	0	99.8	2026174	LR216062.1
Neisseria gonorrhoeae strain 10610 chromosome, complete genome	Streptococcus pneumoniae	4676	4676	100%	0	99.8	2039955	CP079922.1
Neisseria gonorrhoeae strain 10524 chromosome, complete genome	Streptococcus pneumoniae	4676	4676	100%	0	99.8	2025465	LR216037.1

Neisseria gonorrhoeae strain 10794 chromosome, complete genome	Streptococcus pneumoniae	4676	4676	100%	0	99.8	2220550	CP067363.1
Neisseria gonorrhoeae strain 10531 chromosome, complete genome	Streptococcus pneumoniae	4671	4671	100%	0	99.76	2170541	AP026922.1
Neisseria gonorrhoeae strain 10795 chromosome, complete genome	Streptococcus pneumoniae	4671	4671	100%	0	99.76	2141530	CP035249.1
Neisseria gonorrhoeae strain 10531 chromosome, complete genome	Streptococcus pneumoniae	4671	4671	100%	0	99.76	2120990	CP035264.1
Neisseria gonorrhoeae strain 10708 chromosome, complete genome	Streptococcus pneumoniae	4671	4671	100%	0	99.76	2025152	CP132060.1
Neisseria gonorrhoeae strain 10723 chromosome, complete genome	Streptococcus pneumoniae	4671	4671	100%	0	99.76	2024947	CP051650.1
Neisseria gonorrhoeae strain 10328 chromosome, complete genome	Streptococcus pneumoniae	4671	4671	100%	0	99.76	2150823	CP155535.1

Table S2 Conservativeness analysis of antigenic sequences (PepO) was found in NCBI to be present in the following 100 genomes, with 99.74-100% nucleic acid identity).

Description	Scientific Name	Max Score	Total Score	Query Cover	E value	Per. ident	Acc. Len	Accession
Streptococcus pneumoniae strain 2245STDY5775874 genome assembly, chromosome: 1	Streptococcus pneumoniae	3496	3496	100%	0	100	2105368	LR216032.1
Streptococcus pneumoniae strain TVO_1901923 chromosome, complete genome	Streptococcus pneumoniae	3496	3496	100%	0	100	2116769	CP035262.1
Streptococcus pneumoniae strain 947 genome assembly, chromosome: 1	Streptococcus pneumoniae	3496	3496	100%	0	100	2109624	LR129841.1
Streptococcus pneumoniae TIGR4	Streptococcus pneumoniae	3496	3496	100%	0	100	2153837	CP155539.1

chromosome, complete genome	TIGR4							
Streptococcus pneumoniae strain FC2 chromosome, complete genome	Streptococcus pneumoniae	3496	3496	100%	0	100	2131566	CP168260.1
Streptococcus pneumoniae strain 2245STDY5775485 genome assembly, chromosome: 1	Streptococcus pneumoniae	3496	3496	100%	0	100	2061600	LR216026.1
Streptococcus pneumoniae INV200 genome	Streptococcus pneumoniae INV200	3496	3496	100%	0	100	2093317	FQ312029.1
Streptococcus pneumoniae Xen35, complete genome	Streptococcus pneumoniae	3496	3496	100%	0	100	2168552	CP025256.1
Streptococcus pneumoniae strain TVO_TIGR4 chromosome, complete genome	Streptococcus pneumoniae	3496	3496	100%	0	100	2153840	CP035239.1
Streptococcus pneumoniae strain 15H4024 chromosome, complete genome	Streptococcus pneumoniae	3496	3496	100%	0	100	2187550	CP137107.1
Streptococcus pneumoniae strain TVO_1902282 chromosome, complete genome	Streptococcus pneumoniae	3496	3496	100%	0	100	2103577	CP035236.1
Streptococcus pneumoniae strain M26368 chromosome, complete genome	Streptococcus pneumoniae	3496	3496	100%	0	100	2109726	CP031246.1
Streptococcus pneumoniae strain EF3030 chromosome, complete genome	Streptococcus pneumoniae	3496	3496	100%	0	100	2101621	CP035897.1
Streptococcus pneumoniae strain 4559 genome assembly, chromosome: 1	Streptococcus pneumoniae	3496	3496	100%	0	100	2150981	LR595848.1
Streptococcus pneumoniae strain 2245STDY6105839 genome assembly, chromosome: 1	Streptococcus pneumoniae	3496	3496	100%	0	100	2086119	LR216063.1
Streptococcus pneumoniae strain BHN97x chromosome, complete genome	Streptococcus pneumoniae	3496	3496	100%	0	100	2125517	CP025076.1
Streptococcus pneumoniae strain 02H2025 chromosome, complete genome	Streptococcus pneumoniae	3496	3496	100%	0	100	2163419	CP137112.1

Streptococcus pneumoniae strain TVO_1901922 chromosome, complete genome	Streptococcus pneumoniae	3496	3496	100%	0	100	2145531	CP035263.1
Streptococcus pneumoniae strain 2245STDY5775553 genome assembly, chromosome: 1	Streptococcus pneumoniae	3496	3496	100%	0	100	2143696	LR536833.1
Streptococcus pneumoniae strain 2245STDY5775603 genome assembly, chromosome: 1	Streptococcus pneumoniae	3496	3496	100%	0	100	2107041	LR216036.1
Streptococcus pneumoniae strain EF3030 chromosome	Streptococcus pneumoniae	3496	3496	100%	0	100	2209198	CP026549.1
Streptococcus pneumoniae TIGR4, complete genome	Streptococcus pneumoniae TIGR4	3496	3496	100%	0	100	2160842	AE005672.3
Streptococcus pneumoniae TIGR4 chromosome, complete genome	Streptococcus pneumoniae TIGR4	3496	3496	100%	0	100	2153849	CP089948.1
Streptococcus pneumoniae strain BC1 chromosome, complete genome	Streptococcus pneumoniae	3491	3491	100%	0	99.95	2136743	CP113266.1
Streptococcus pneumoniae strain NP7536 chromosome, complete genome	Streptococcus pneumoniae	3491	3491	100%	0	99.95	2062504	CP090887.1
Streptococcus pneumoniae strain L1900 chromosome, complete genome	Streptococcus pneumoniae	3491	3491	100%	0	99.95	2025152	CP132060.1
Streptococcus pneumoniae strain B1900 chromosome, complete genome	Streptococcus pneumoniae	3491	3491	100%	0	99.95	2024947	CP051650.1
Streptococcus pneumoniae PZ900700097 DNA, complete genome	Streptococcus pneumoniae	3491	3491	100%	0	99.95	2091173	AP026925.1
Streptococcus pneumoniae PZ900701549 DNA, complete genome	Streptococcus pneumoniae	3491	3491	100%	0	99.95	2138339	AP026932.1
Streptococcus pneumoniae strain CH2241 chromosome, complete genome	Streptococcus pneumoniae	3491	3491	100%	0	99.95	2062088	CP090882.1

Streptococcus pneumoniae strain 20614-6 chromosome, complete genome	Streptococcus pneumoniae	3491	3491	100%	0	99.95	2069036	CP137115.1
Streptococcus pneumoniae strain H1900 chromosome, complete genome	Streptococcus pneumoniae	3491	3491	100%	0	99.95	2025162	CP132059.1
Streptococcus pneumoniae strain 16H2041 chromosome, complete genome	Streptococcus pneumoniae	3491	3491	100%	0	99.95	2076043	CP137110.1
Streptococcus pneumoniae strain ND6401 chromosome, complete genome	Streptococcus pneumoniae	3491	3491	100%	0	99.95	2061648	CP090885.1
Streptococcus pneumoniae SPN034156 draft genome	Streptococcus pneumoniae SPN034156	3491	3491	100%	0	99.95	2024476	FQ312045.1
Streptococcus pneumoniae DNA, nearly complete genome, strain: KK0381	Streptococcus pneumoniae	3491	3491	100%	0	99.95	2158944	AP018043.1
Streptococcus pneumoniae strain PT8465 chromosome, complete genome	Streptococcus pneumoniae	3491	3491	100%	0	99.95	2046177	CP090888.1
Streptococcus pneumoniae strain MDR-MM-0720-0522 chromosome	Streptococcus pneumoniae	3491	3491	100%	0	99.95	2220550	CP067363.1
Streptococcus pneumoniae strain NCTC11902 genome assembly, chromosome: 1	Streptococcus pneumoniae	3485	3485	100%	0	99.89	2093242	LS483417.1
Streptococcus pneumoniae ST556, complete genome	Streptococcus pneumoniae ST556	3485	3485	100%	0	99.89	2150813	CP003357.2
Streptococcus pneumoniae MDRSPN001 DNA, complete genome	Streptococcus pneumoniae	3485	3485	100%	0	99.89	2045062	AP018391.1
Streptococcus pneumoniae strain 2245STDY6020210 genome assembly, chromosome: 1	Streptococcus pneumoniae	3485	3485	100%	0	99.89	2097910	LR216043.1
Streptococcus pneumoniae strain 2245STDY5605535 genome assembly, chromosome: 1	Streptococcus pneumoniae	3485	3485	100%	0	99.89	2053788	LR216016.1
Streptococcus pneumoniae HU-OH DNA, complete genome	Streptococcus pneumoniae	3485	3485	100%	0	99.89	2058492	AP018937.1

Streptococcus pneumoniae strain 15P3054 chromosome, complete genome	Streptococcus pneumoniae	3485	3485	100%	0	99.89	2072679	CP137114.1
Streptococcus pneumoniae strain TVO_1901927 chromosome, complete genome	Streptococcus pneumoniae	3485	3485	100%	0	99.89	2118571	CP035258.1
Streptococcus pneumoniae strain TVO_Taiwan19F-14 chromosome, complete genome	Streptococcus pneumoniae	3485	3485	100%	0	99.89	2112180	CP035237.1
Streptococcus pneumoniae Taiwan19F-14, complete genome	Streptococcus pneumoniae Taiwan19F-14	3485	3485	100%	0	99.89	2112148	CP000921.1
Streptococcus pneumoniae strain TVO_1901926 chromosome, complete genome	Streptococcus pneumoniae	3485	3485	100%	0	99.89	2079593	CP035259.1
Streptococcus pneumoniae PZ900700406 DNA, complete genome	Streptococcus pneumoniae	3485	3485	100%	0	99.89	2098820	AP026928.1
Streptococcus pneumoniae strain 2245STDY5605682 genome assembly, chromosome: 1	Streptococcus pneumoniae	3485	3485	100%	0	99.89	2129739	LR216018.1
Streptococcus pneumoniae strain 6_2F1 chromosome, complete genome	Streptococcus pneumoniae	3485	3485	100%	0	99.89	2069030	CP169564.1
Streptococcus pneumoniae strain NT_110_58, complete genome	Streptococcus pneumoniae	3485	3485	100%	0	99.89	2287774	CP007593.1
Streptococcus pneumoniae strain NP7513 chromosome, complete genome	Streptococcus pneumoniae	3485	3485	100%	0	99.89	2057101	CP090886.1
Streptococcus pneumoniae strain 2245STDY6093044 genome assembly, chromosome: 1	Streptococcus pneumoniae	3485	3485	100%	0	99.89	2079558	LR216049.1
Streptococcus pneumoniae strain 2245STDY5562562 genome assembly, chromosome: 1	Streptococcus pneumoniae	3485	3485	100%	0	99.89	2051563	LR216025.1
Streptococcus pneumoniae strain 2245STDY6179186 genome assembly,	Streptococcus pneumoniae	3485	3485	100%	0	99.89	2019985	LR216066.1

chromosome: 1								
Streptococcus pneumoniae strain TVO_1901932 chromosome, complete genome	Streptococcus pneumoniae	3480	3480	100%	0	99.84	2118426	CP035254.1
Streptococcus pneumoniae strain 2018C08-270 chromosome, complete genome	Streptococcus pneumoniae	3480	3480	100%	0	99.84	2128646	CP131707.1
Streptococcus pneumoniae strain TVO_1901933 chromosome	Streptococcus pneumoniae	3480	3480	100%	0	99.84	2130843	CP035253.1
Streptococcus pneumoniae strain 2245STDY6106337 genome assembly, chromosome: 1	Streptococcus pneumoniae	3480	3480	100%	0	99.84	2082222	LR216051.1
Streptococcus pneumoniae strain 2245STDY6092581 genome assembly, chromosome: 1	Streptococcus pneumoniae	3480	3480	100%	0	99.84	2104343	LR216042.1
Streptococcus pneumoniae isolate SA_GPS_SP505-sc-1895675 genome assembly, chromosome: 1	Streptococcus pneumoniae	3474	3474	100%	0	99.79	2103862	LR216035.1
Streptococcus pneumoniae strain ST62 chromosome, complete genome	Streptococcus pneumoniae	3474	3474	100%	0	99.79	2027961	CP136899.1
Streptococcus pneumoniae strain 17023 chromosome, complete genome	Streptococcus pneumoniae	3474	3474	100%	0	99.79	2110175	CP137111.1
Streptococcus pneumoniae strain 21011 chromosome, complete genome	Streptococcus pneumoniae	3474	3474	100%	0	99.79	2100423	CP139860.1
Streptococcus pneumoniae strain LYP chromosome, complete genome	Streptococcus pneumoniae	3474	3474	100%	0	99.79	2200466	CP137100.1
Streptococcus pneumoniae strain Hu15 chromosome	Streptococcus pneumoniae	3474	3474	100%	0	99.79	2147991	CP020551.1
Streptococcus pneumoniae AP200, complete genome	Streptococcus pneumoniae AP200	3474	3474	100%	0	99.79	2130580	CP002121.1
Streptococcus pneumoniae strain TVO_1901948 chromosome, complete	Streptococcus pneumoniae	3474	3474	100%	0	99.79	2001615	CP035241.1

genome								
Streptococcus pneumoniae strain TVO_1901925 chromosome, complete genome	Streptococcus pneumoniae	3474	3474	100%	0	99.79	2000117	CP035260.1
Streptococcus pneumoniae strain 2245STDY6178826 genome assembly, chromosome: 1	Streptococcus pneumoniae	3474	3474	100%	0	99.79	2154542	LR216061.1
Streptococcus pneumoniae strain 20824-4 chromosome, complete genome	Streptococcus pneumoniae	3474	3474	100%	0	99.79	2124888	CP137099.1
Streptococcus pneumoniae strain 2245STDY6092613 genome assembly, chromosome: 1	Streptococcus pneumoniae	3474	3474	100%	0	99.79	2129170	LR216046.1
Streptococcus pneumoniae strain 105_Kz chromosome, complete genome	Streptococcus pneumoniae	3474	3474	100%	0	99.79	2036779	CP125291.1
Streptococcus pneumoniae strain 2245STDY5868782 genome assembly, chromosome: 1	Streptococcus pneumoniae	3474	3474	100%	0	99.79	2081497	LR216033.1
Streptococcus pneumoniae strain 2245STDY5775545 genome assembly, chromosome: 1	Streptococcus pneumoniae	3474	3474	100%	0	99.79	2047964	LR216030.1
Streptococcus pneumoniae Sp943 DNA, complete genome	Streptococcus pneumoniae	3474	3474	100%	0	99.79	2104196	AP031341.1
Streptococcus pneumoniae Pne2 DNA, complete genome	Streptococcus pneumoniae	3469	3469	100%	0	99.74	2194831	AP028603.1
Streptococcus pneumoniae strain TVO_1901931 chromosome	Streptococcus pneumoniae	3469	3469	100%	0	99.74	2156376	CP035255.1
Streptococcus pneumoniae strain 566 chromosome, complete genome	Streptococcus pneumoniae	3469	3469	100%	0	99.74	2124184	CP046358.1
Streptococcus pneumoniae PZ900700027 DNA, complete genome	Streptococcus pneumoniae	3469	3469	100%	0	99.74	2156586	AP026921.1
Streptococcus pneumoniae strain 16H2017-2 chromosome, complete	Streptococcus pneumoniae	3469	3469	100%	0	99.74	2081720	CP139861.1

genome								
Streptococcus pneumoniae 16P28 DNA, complete genome	Streptococcus pneumoniae	3469	3469	100%	0	99.74	2144693	AP031394.1
Streptococcus pneumoniae isolate GPS_ZA_821-sc-1950967 genome assembly, chromosome: 1	Streptococcus pneumoniae	3469	3469	100%	0	99.74	2133955	LR536845.1
Streptococcus pneumoniae Sp575 DNA, complete genome	Streptococcus pneumoniae	3469	3469	100%	0	99.74	2138474	AP031340.1
Streptococcus pneumoniae PZ900700063 DNA, complete genome	Streptococcus pneumoniae	3469	3469	100%	0	99.74	2218836	AP026924.1
Streptococcus pneumoniae strain NP1 chromosome, complete genome	Streptococcus pneumoniae	3469	3469	100%	0	99.74	2310574	CP113114.1
Streptococcus pneumoniae Pne3 DNA, complete genome	Streptococcus pneumoniae	3469	3469	100%	0	99.74	2194794	AP028604.1
Streptococcus pneumoniae PF1 DNA, complete genome	Streptococcus pneumoniae	3469	3469	100%	0	99.74	2054748	AP028601.1
Streptococcus pneumoniae strain BHN418 chromosome, complete genome	Streptococcus pneumoniae	3469	3469	100%	0	99.74	2114355	CP155538.1
Streptococcus pneumoniae PZ900700012 DNA, complete genome	Streptococcus pneumoniae	3469	3469	100%	0	99.74	2148358	AP026919.1
Streptococcus pneumoniae strain NP2 chromosome, complete genome	Streptococcus pneumoniae	3469	3469	100%	0	99.74	2292804	CP113115.1
Streptococcus pneumoniae Sep2 DNA, complete genome	Streptococcus pneumoniae	3469	3469	100%	0	99.74	2143010	AP028607.1
Streptococcus pneumoniae strain 310 chromosome, complete genome	Streptococcus pneumoniae	3469	3469	100%	0	99.74	2121469	CP046354.1
Streptococcus pneumoniae 16P29 DNA, complete genome	Streptococcus pneumoniae	3469	3469	100%	0	99.74	2145671	AP031395.1
Streptococcus pneumoniae PZ900700054 DNA, complete genome	Streptococcus pneumoniae	3469	3469	100%	0	99.74	2099632	AP026923.1

Streptococcus pneumoniae strain 16H2092 chromosome, complete genome	Streptococcus pneumoniae	3469	3469	100%	0	99.74	2121940	CP137105.1

Table S3 Conservativeness analysis of antigenic sequences (SPD_1609) was found in NCBI to be present in the following 100 genomes, with 95.88-100% nucleic acid identity).

Description	Scientific Name	Max Score	Total Score	Query Cover	E value	Per. ident	Acc. Len	Accession
Streptococcus pneumoniae TIGR4 chromosome, complete genome	Streptococcus pneumoniae TIGR4	1973	1973	100%	0	100	2153837	CP155539.1
Streptococcus pneumoniae Xen35, complete genome	Streptococcus pneumoniae	1973	1973	100%	0	100	2168552	CP025256.1
Streptococcus pneumoniae strain TVO_TIGR4 chromosome, complete genome	Streptococcus pneumoniae	1973	1973	100%	0	100	2153840	CP035239.1
Streptococcus pneumoniae TIGR4, complete genome	Streptococcus pneumoniae TIGR4	1973	1973	100%	0	100	2160842	AE005672.3
Streptococcus pneumoniae TIGR4 chromosome, complete genome	Streptococcus pneumoniae TIGR4	1973	1973	100%	0	100	2153849	CP089948.1
Streptococcus pneumoniae strain 20155336 chromosome, complete genome	Streptococcus pneumoniae	1940	1940	100%	0	99.44	2000745	CP154876.1
Streptococcus pneumoniae strain TVO_1901921 chromosome, complete genome	Streptococcus pneumoniae	1940	1940	100%	0	99.44	2120990	CP035264.1
Streptococcus pneumoniae strain 2245STDY6020221 genome assembly,	Streptococcus pneumoniae	1940	1940	100%	0	99.44	2066149	LR216045.1

chromosome: 1								
Streptococcus pneumoniae strain PZ900700608 chromosome, complete genome	Streptococcus pneumoniae	1940	1940	100%	0	99.44	2210224	CP060156.1
Streptococcus pneumoniae strain TVO_1901920 chromosome, complete genome	Streptococcus pneumoniae	1940	1940	100%	0	99.44	2084495	CP035265.1
Streptococcus pneumoniae strain 20234295 chromosome, complete genome	Streptococcus pneumoniae	1940	1940	100%	0	99.44	1999917	CP154877.1
Streptococcus pneumoniae strain 2245STDY5983173 genome assembly, chromosome: 1	Streptococcus pneumoniae	1934	1934	100%	0	99.34	2097748	LR216034.1
Streptococcus pneumoniae strain TVO_1901945 chromosome, complete genome	Streptococcus pneumoniae	1923	1923	100%	0	99.16	2098679	CP035244.1
Streptococcus pneumoniae strain 2245STDY6106372 genome assembly, chromosome: 1	Streptococcus pneumoniae	1917	1917	100%	0	99.06	2102932	LR216054.1
Streptococcus pneumoniae ST556, complete genome	Streptococcus pneumoniae ST556	1906	1906	100%	0	98.88	2150813	CP003357.2
Streptococcus pneumoniae strain 3641/15 chromosome, complete genome	Streptococcus pneumoniae	1906	1906	100%	0	98.88	2069241	CP091450.1
Streptococcus pneumoniae strain 3238/09 chromosome, complete genome	Streptococcus pneumoniae	1906	1906	100%	0	98.88	2103144	CP091451.1
Streptococcus pneumoniae strain 2014C08-275 chromosome, complete genome	Streptococcus pneumoniae	1906	1906	100%	0	98.88	2093106	CP131711.1
Streptococcus pneumoniae strain RMV7 genome assembly, chromosome: 1	Streptococcus pneumoniae	1906	1906	100%	0	98.88	2112955	OV904788.1
Streptococcus pneumoniae MDRSPN001 DNA, complete genome	Streptococcus pneumoniae	1906	1906	100%	0	98.88	2045062	AP018391.1

Streptococcus pneumoniae strain SP007 chromosome, complete genome	Streptococcus pneumoniae	1906	1906	100%	0	98.88	2088456	CP096809.1
Streptococcus pneumoniae strain TVO_Taiwan19F-14 chromosome, complete genome	Streptococcus pneumoniae	1906	1906	100%	0	98.88	2112180	CP035237.1
Streptococcus pneumoniae Taiwan19F-14, complete genome	Streptococcus pneumoniae Taiwan19F-14	1906	1906	100%	0	98.88	2112148	CP000921.1
Streptococcus pneumoniae strain 2016S11-245 chromosome, complete genome	Streptococcus pneumoniae	1906	1906	100%	0	98.88	2090920	CP131709.1
Streptococcus pneumoniae strain 21334 chromosome, complete genome	Streptococcus pneumoniae	1906	1906	100%	0	98.88	2109372	CP137103.1
Streptococcus pneumoniae strain SP61 chromosome, complete genome	Streptococcus pneumoniae	1906	1906	100%	0	98.88	2071812	CP018137.1
Streptococcus pneumoniae A026 genome	Streptococcus pneumoniae A026	1906	1906	100%	0	98.88	2091879	CP006844.1
Streptococcus pneumoniae strain 19A-19087 chromosome, complete genome	Streptococcus pneumoniae	1906	1906	100%	0	98.88	2159119	CP071916.1
Streptococcus pneumoniae strain OXT chromosome, complete genome	Streptococcus pneumoniae	1906	1906	100%	0	98.88	2136646	CP137108.1
Streptococcus pneumoniae strain SWU02, complete genome	Streptococcus pneumoniae	1906	1906	100%	0	98.88	2092148	CP018347.1
Streptococcus pneumoniae strain 19A-19339 chromosome, complete genome	Streptococcus pneumoniae	1906	1906	100%	0	98.88	2196665	CP071917.1
Streptococcus pneumoniae strain 19A-ST320_99-176 chromosome, complete genome	Streptococcus pneumoniae	1906	1906	100%	0	98.88	2089994	CP063829.1
Streptococcus pneumoniae strain 19A-19343 chromosome, complete genome	Streptococcus pneumoniae	1906	1906	100%	0	98.88	2195807	CP071918.1

Streptococcus pneumoniae strain SP64 chromosome, complete genome	Streptococcus pneumoniae	1906	1906	100%	0	98.88	2073113	CP018138.1
Streptococcus pneumoniae strain LM chromosome, complete genome	Streptococcus pneumoniae	1906	1906	100%	0	98.88	2136646	CP137104.1
Streptococcus pneumoniae strain SN36255 chromosome, complete genome	Streptococcus pneumoniae	1906	1906	100%	0	98.88	2088681	CP155531.1
Streptococcus pneumoniae TCH8431/19A, complete genome	Streptococcus pneumoniae TCH8431/19A	1906	1906	100%	0	98.88	2088772	CP001993.1
Streptococcus pneumoniae strain SPN XDR SMC1710-32 chromosome, complete genome	Streptococcus pneumoniae	1901	1901	100%	0	98.78	2057144	CP025838.1
Streptococcus pneumoniae PZ900700012 DNA, complete genome	Streptococcus pneumoniae	1901	1901	100%	0	98.78	2148358	AP026919.1
Streptococcus pneumoniae strain 11A chromosome, complete genome	Streptococcus pneumoniae	1901	1901	100%	0	98.78	2079194	CP018838.1
Streptococcus pneumoniae strain SN75752 chromosome, complete genome	Streptococcus pneumoniae	1895	1895	100%	0	98.69	2151943	CP089949.1
Streptococcus pneumoniae strain 2245STDY5775874 genome assembly, chromosome: 1	Streptococcus pneumoniae	1890	1890	100%	0	98.6	2105368	LR216032.1
Streptococcus pneumoniae strain 2245STDY5775485 genome assembly, chromosome: 1	Streptococcus pneumoniae	1884	1884	100%	0	98.5	2061600	LR216026.1
Streptococcus pneumoniae strain 2245STDY5562369 genome assembly, chromosome: 1	Streptococcus pneumoniae	1845	1845	100%	0	97.85	2103319	LR216015.1
Streptococcus pneumoniae strain TVO_1901923 chromosome, complete genome	Streptococcus pneumoniae	1840	1840	100%	0	97.75	2116769	CP035262.1
Streptococcus pneumoniae strain TVO_1901931 chromosome	Streptococcus pneumoniae	1735	1735	100%	0	95.98	2156376	CP035255.1

Streptococcus pneumoniae strain TVO_1901930 chromosome, complete genome	Streptococcus pneumoniae	1735	1735	100%	0	95.98	2223053	CP035256.1
Streptococcus pneumoniae strain 4041STDY6836169 genome assembly, chromosome: 1	Streptococcus pneumoniae	1735	1735	100%	0	95.98	2145551	LS483523.1
Streptococcus pneumoniae strain BHN418 chromosome, complete genome	Streptococcus pneumoniae	1735	1735	100%	0	95.98	2114355	CP155538.1
Streptococcus pneumoniae strain 27_Kz chromosome	Streptococcus pneumoniae	1735	1735	100%	0	95.98	2083628	CP126249.1
Streptococcus pneumoniae strain CP2215 chromosome	Streptococcus pneumoniae	1735	1735	100%	0	95.98	2122840	CP028436.1
Streptococcus pneumoniae strain 2008C09-276 chromosome, complete genome	Streptococcus pneumoniae	1729	1729	100%	0	95.88	2145516	CP131713.1
Streptococcus pneumoniae strain AUSMDU00010538 chromosome, complete genome	Streptococcus pneumoniae	1729	1729	100%	0	95.88	2090792	CP045931.1
Streptococcus pneumoniae strain SP264 chromosome, complete genome	Streptococcus pneumoniae	1729	1729	100%	0	95.88	2185927	CP155532.1
Streptococcus pneumoniae Pne2 DNA, complete genome	Streptococcus pneumoniae	1729	1729	100%	0	95.88	2194831	AP028603.1
Streptococcus pneumoniae strain ST62 chromosome, complete genome	Streptococcus pneumoniae	1729	1729	100%	0	95.88	2027961	CP136899.1
Streptococcus pneumoniae PZ900700119 DNA, complete genome	Streptococcus pneumoniae	1729	1729	100%	0	95.88	2190915	AP026926.1
Streptococcus pneumoniae Sp525 DNA, complete genome	Streptococcus pneumoniae	1729	1729	100%	0	95.88	2164261	AP031339.1
Streptococcus pneumoniae strain 2245STDY5562351 genome assembly, chromosome: 1	Streptococcus pneumoniae	1729	1729	100%	0	95.88	1985764	LR216020.1

Streptococcus pneumoniae strain TVO_1901928 chromosome, complete genome	Streptococcus pneumoniae	1729	1729	100%	0	95.88	2159294	CP035257.1
Streptococcus pneumoniae strain TVO_1901932 chromosome, complete genome	Streptococcus pneumoniae	1729	1729	100%	0	95.88	2118426	CP035254.1
Streptococcus pneumoniae PZ900700027 DNA, complete genome	Streptococcus pneumoniae	1729	1729	100%	0	95.88	2156586	AP026921.1
Streptococcus pneumoniae strain DCC1476 chromosome, complete genome	Streptococcus pneumoniae	1729	1729	100%	0	95.88	2157375	CP155534.1
Streptococcus pneumoniae strain GA40410 chromosome	Streptococcus pneumoniae	1729	1729	100%	0	95.88	2120188	CP118282.1
Streptococcus pneumoniae Sp524 DNA, complete genome	Streptococcus pneumoniae	1729	1729	100%	0	95.88	2157651	AP031338.1
Streptococcus pneumoniae strain 2245STDY6106635 genome assembly, chromosome: 1	Streptococcus pneumoniae	1729	1729	100%	0	95.88	2126260	LR536837.1
Streptococcus pneumoniae strain Hu15 chromosome	Streptococcus pneumoniae	1729	1729	100%	0	95.88	2147991	CP020551.1
Streptococcus pneumoniae strain 16H2017-2 chromosome, complete genome	Streptococcus pneumoniae	1729	1729	100%	0	95.88	2081720	CP139861.1
Streptococcus pneumoniae PZ900701541 DNA, complete genome	Streptococcus pneumoniae	1729	1729	100%	0	95.88	2106979	AP026931.1
Streptococcus pneumoniae strain TVO_1901936 chromosome, complete genome	Streptococcus pneumoniae	1729	1729	100%	0	95.88	2139846	CP038252.1
Streptococcus pneumoniae strain 4041STDY6836167 genome assembly, chromosome: 1	Streptococcus pneumoniae	1729	1729	100%	0	95.88	2131442	LS483448.1
Streptococcus pneumoniae isolate GPS_HK_21-sc-2296565 genome assembly, chromosome: 1	Streptococcus pneumoniae	1729	1729	100%	0	95.88	2121917	LR216058.1

Streptococcus pneumoniae strain FDAARGOS_1508 chromosome, complete genome	Streptococcus pneumoniae	1729	1729	100%	0	95.88	2116633	CP083627.1
Streptococcus pneumoniae strain 2245STDY6092834 genome assembly, chromosome: 1	Streptococcus pneumoniae	1729	1729	100%	0	95.88	2107358	LR216048.1
Streptococcus pneumoniae Pne3 DNA, complete genome	Streptococcus pneumoniae	1729	1729	100%	0	95.88	2194794	AP028604.1
Streptococcus pneumoniae strain 105_Kz chromosome, complete genome	Streptococcus pneumoniae	1729	1729	100%	0	95.88	2036779	CP125291.1
Streptococcus pneumoniae strain BHN97x chromosome, complete genome	Streptococcus pneumoniae	1729	1729	100%	0	95.88	2125517	CP025076.1
Streptococcus pneumoniae strain TVO_1901934 chromosome, complete genome	Streptococcus pneumoniae	1729	1729	100%	0	95.88	2165373	CP035252.1
Streptococcus pneumoniae PZ900700009 DNA, complete genome	Streptococcus pneumoniae	1729	1729	100%	0	95.88	2090609	AP026918.1
Streptococcus pneumoniae strain 2008C04-309 chromosome, complete genome	Streptococcus pneumoniae	1729	1729	100%	0	95.88	2146355	CP131714.1
Streptococcus pneumoniae strain TL7/1993 chromosome, complete genome	Streptococcus pneumoniae	1729	1729	100%	0	95.88	2156500	CP156625.1
Streptococcus pneumoniae strain 2014S11-203 chromosome, complete genome	Streptococcus pneumoniae	1729	1729	100%	0	95.88	2140064	CP131710.1
Streptococcus pneumoniae strain 335 chromosome, complete genome	Streptococcus pneumoniae	1729	1729	100%	0	95.88	2221315	CP026670.1
Streptococcus pneumoniae strain 2008C09-280 chromosome, complete genome	Streptococcus pneumoniae	1729	1729	100%	0	95.88	2207415	CP131712.1
Streptococcus pneumoniae strain 2016C10-332 chromosome, complete	Streptococcus	1729	1729	100%	0	95.88	2175793	CP131708.1

genome	pneumoniae							
Streptococcus pneumoniae Sep3 DNA, complete genome	Streptococcus pneumoniae	1729	1729	100%	0	95.88	2039063	AP028608.1
Streptococcus pneumoniae strain 2018N21-288 chromosome, complete genome	Streptococcus pneumoniae	1729	1729	100%	0	95.88	2109177	CP131706.1
Streptococcus pneumoniae strain TVO_1901941 chromosome, complete genome	Streptococcus pneumoniae	1729	1729	100%	0	95.88	2050463	CP035246.1
Streptococcus pneumoniae strain 2245STDY5699131 genome assembly, chromosome: 1	Streptococcus pneumoniae	1729	1729	100%	0	95.88	2084323	LR216022.1
Streptococcus pneumoniae 21P20 DNA, complete genome	Streptococcus pneumoniae	1729	1729	100%	0	95.88	2107654	AP028877.1
Streptococcus pneumoniae strain GA43265 chromosome	Streptococcus pneumoniae	1729	1729	100%	0	95.88	2122254	CP118281.1
Streptococcus pneumoniae strain TVO_1901933 chromosome	Streptococcus pneumoniae	1729	1729	100%	0	95.88	2130843	CP035253.1
Streptococcus pneumoniae strain 2006C08-243 chromosome, complete genome	Streptococcus pneumoniae	1729	1729	100%	0	95.88	2138456	CP131715.1
Streptococcus pneumoniae strain PT8465 chromosome, complete genome	Streptococcus pneumoniae	1729	1729	100%	0	95.88	2046177	CP090888.1
Streptococcus pneumoniae strain ATCC 700671 chromosome, complete genome	Streptococcus pneumoniae	1729	1729	100%	0	95.88	2116814	CP099641.1
Streptococcus pneumoniae strain TVO_1901935 chromosome, complete genome	Streptococcus pneumoniae	1729	1729	100%	0	95.88	2098476	CP035251.1
Streptococcus pneumoniae strain GA43265 chromosome	Streptococcus pneumoniae	1729	1729	100%	0	95.88	2108048	CP118280.1
Streptococcus pneumoniae strain Hu17 chromosome	Streptococcus pneumoniae	1729	1729	100%	0	95.88	2148350	CP020549.1
